# Marketing of oral nicotine pouches on Malaysian social media and e-commerce websites

**DOI:** 10.18332/tid/219213

**Published:** 2026-05-29

**Authors:** Chandrashekhar T. Sreeramareddy, Pei Kuan Lai, Divya Reshmi

**Affiliations:** 1 Department of Public Health and Community Medicine, IMU University, Kuala Lumpur, Malaysia; 2 Centre for Translational Research Institute for Research Development and Innovation, IMU University, Kuala Lumpur, Malaysia

**Keywords:** oral nicotine pouches, marketing, Malaysia

## Abstract

**INTRODUCTION:**

There are reports of oral nicotine pouches (ONPs) being marketed in high-income countries. The aim of this study was to report online promotions and marketing claims about ONPs in Malaysia, which currently does not regulate nicotine pouches.

**METHODS:**

We did a content analysis of ONPs sold on e-stores. We searched the terms: ‘Velo’, ‘Zyn’, ‘Onz’, ‘nicotine pouch’, and ‘oral nicotine pouch’ on Google Malaysia, Facebook, Reddit, TikTok, and Instagram during March–April 2024. Three trained coders independently coded the e-stores, using a codebook developed based on previous research. Visual and textual information about brands, prices, discounts, flavors, nicotine strength, and marketing claims, was collected. Results are presented in both descriptive and free text formats.

**RESULTS:**

A sample of 41 e-stores was analyzed. ONP marketing was present on TikTok and e-commerce stores. Brands such as ‘Velo’, ‘Zyn’, and ‘Boltbe’ offered discounts, free deliveries, and an array of flavors, with nicotine strengths of 2–50 mg. All brands displayed nicotine strength (mg). Only 51% of the e-stores explicitly stated that the product contained ‘nicotine’, and 5% disclosed that nicotine is addictive; 41% of the stores displayed ONPs as ‘tobacco-free’. About half of the e-stores had marketing claims of ‘product appeal’ (56%), ‘convenient to use anywhere, everywhere’ (46%), and ‘smoking cessation’ (49%). Age verifications and identification proofs were not available at all, whereas health warnings against usage by minors, non-smokers, and pregnant women, were present on only 5%.

**CONCLUSIONS:**

Online marketing and sales promotions were present on e-commerce websites and some social media platforms. The range of flavors and strengths, sales promotions, marketing claims such as appeal and convenience of use, the lack of nicotine disclaimers, and age verification were noted. Surveillance of ONPs is needed to inform regulatory policy.

## INTRODUCTION

The last few decades have witnessed changing patterns of tobacco products, with the introduction of non-combustible nicotine-containing products claimed to be less harmful than traditional combustible tobacco products^1^. Oral nicotine pouches (ONPs) represent a reduced-risk substitute for oral tobacco products, as they have lower toxicants^2^. ONPs are to be held between the user’s upper lip and gums and were first introduced in Sweden, the US, and the UK in 2019^3^. Its market size has increased in the US and elsewhere^4,5^. However, currently, ONP is most prevalent among individuals who smoke cigarettes^6,7^. Though ONPs are not known to be sold in all countries, only a few countries currently regulate them^8^. ONPs can serve as nicotine replacement products with potential benefit to those who smoke cigarettes as a harm-reduction tool^9^ or possibly even as a cessation tool^10^. However, the public health potential of ONP should be carefully weighed against its emergent unintended consequences^11^ of dual use of ONPs with cigarette smoking, leading to a higher level of addiction and health consequences^11^. Emergence of flavored ONPs^12^ would also lead to uptake of ONPs among nicotine-naïve adults and the youth^13^, and have a potential impact of ONPs on oral health^14^.

Online marketing of ONP has been well-reported via social media^15^, the internet, traditional media^16^ and other media^17,18^ in high-income countries (HICs). There is a report about availability, flavors, flavor chemicals, and nicotine strengths in Pakistan^19,20^. Online marketing and sales of e-cigarettes are known in Malaysia^21^ and other low- and middle-income countries (LMICs)^22^. Newer non-cigarette nicotine products, i.e. e-cigarettes and ONPs, were unregulated in Malaysia until October 2024. In Malaysia, Act 852, known as the Control of Smoking Products for Public Health Act 2024, came into effect on 1 October 2024, and covers both tobacco and vape products^23^. However, Act 852 does not explicitly state whether the regulations cover ONP. The presence of online stores, promotions, and marketing of e-cigarettes is reported in our earlier research^21^, but ONP promotions on online media in Malaysia are not known. Surveillance of online marketing and promotions of novel products, such as ONPs, is important for regulatory policy formulation. The information displayed on the e-commerce stores and marketing strategies used by the online media, is also critical for informing policymakers of possible regulatory strategies targeting these products marketed online. We aimed to examine the presence of online promotions and marketing of ONPs and study the marketing claims, promotional strategies, and product details.

## METHODS

### Study design and sample selection

A content analysis of e-store content was done. The selection criterion was sales of ONPs. Online marketing of ONPs, including the e-stores on e-commerce websites and social media. During March–April 2025, we searched the Google Malaysia website using the terms: ‘nicotine pouch’ and ‘oral nicotine pouch’ and did not identify any physical or online retail stores in Malaysia. We also searched four social media platforms in Malaysia, namely Facebook, Reddit, TikTok, and Instagram, using the same keywords as above. However, our Google search revealed the presence of ONPs on two e-commerce websites, Lazada and Shopee. Within these e-commerce sites, we used the terms ‘nicotine pouch’ and ‘oral nicotine pouches’ to search for the e-stores that sold ONPs. The selection criteria were sales of ONPs. A content analysis was done of the information about ONPs, using a code book that was developed after reviewing the published literature on ONPs^16-18^. Each identified online site for the sale of ONPs is referred to as an e-store.

### Codebook

All three researchers (CTS, LPK, and DR) were involved in searches and coding. To be consistent and for comparability, we adapted the codebook prepared from our previous research^16-18^. The modified code book (Supplementary file Appendix A) was used to manually code the information about the ONP product-related information. The code book included information about the prices, promotions, and instructions to use, flavors, age verification, disclosure about the presence of nicotine, the strength of nicotine, and the disclaimer that nicotine is addictive. The code book also contained information related to health warnings, marketing claims such as health-related, smoking cessation, no secondhand smoke, convenience of use, cheap, product appeal, and any other information of interest that was displayed on the e-stores. For advertisements on Facebook, the number of members on the site was collected.

### Coding method

LPK and DR were trained to use the code book by the principal researcher, CTS. After two rounds of coding by LPK and DR, an inter-rater agreement of 0.82 was achieved. LPK and DR read the content on each e-store to manually extract the data about the descriptors of the LPK and DR, and independently coded each e-store. Any discrepancies were resolved by the principal researcher, CTS. The textual information on the images of e-stores, product pictures, etc., was typed into Microsoft Excel, and the images of e-stores were coded as the presence/absence of each code in a Microsoft Excel sheet. The list of flavors promoted by each brand of ONPs is listed on the e-stores, and the screenshots of the e-stores on e-commerce websites that were coded are provided as online supplements. (Supplementary file Appendices B and C).

### Statistical analysis

The marketing strategies and claims were presented as frequency distributions, and the analysis was done on Microsoft Excel.

### Ethical considerations

This study involved online searches and content analyses of information about ONPs displayed on the e-stores and social media. Ethical approval from the institutional board was not necessary.

## RESULTS

A sample of 41 e-stores was analyzed, i.e. 21 e-stores on Lazada, 14 in Shopee, 4 in TikTok, and two online overseas retail stores from Google search that offered shipping to Malaysia.

### Online marketing on social media

Our search identified various Facebook groups about ONPs originating from non-Malaysian (global) Facebook groups with links to join. But these were not selling ONPs. The members ranged from 4 to 5300. However, there were four instances of online marketing of ONPs on TikTok that were considered e-stores. They contained brand names, nicotine strength, price discounts, and flavors. TikTok contained images (Supplementary file Appendix A) displaying containers of ONP brand names ‘NicPax Pouch’, Mantapp’ being shown by different individuals, and ‘Onz’ and ‘Noma’. The container showed a price of 0.1 RM (1 Malaysian Ringgit about US$0.25) and discounts of 99% and different flavors, namely mango ice, arctic mint, slim cherry, mint freeze, choco dino, harum-mango ice, and Fresh Mint (Supplementary file Appendix B). The strengths were 4 mg and 6 mg without any mention of nicotine in the product (Table 1). Marketing claims such as ‘convenience of use’, i.e. ‘anywhere, everywhere, everywhere’, ‘stop smoking today’, and ‘smoke-free’ were present.

**Table 1:** Detailed information about the products, sellers, manufacturers, prices, and nicotine strengths of ONP products present in 41 e-stores in Malaysia during September–November 2025

*Brand*	*Manufacturer*	*Strength (mg)*	*Price range (RM)*
**LAZADA**			
VELO	British American Tobacco, BAT [7]	4, 4.3, 6, 8, 10, 11, 14, 17, 24	72.09, 78.00
ZYN	Swedish Match – a subsidiary company of Philip Morris International, PMI [16]	15	78.00
SIBERIA	GN Tobacco [11]	22, 24, 28, 33, 43	72.38, 76.74
77	Tobacco Concept Factory [1]	20, 50	74.33, 76.74
VOLT	Swedish Match [12]	6, 10, 14, 16, 18	81.15
PABLO	N.G.P Tobacco [10]	50	95.70
G.3	Swedish Match [4]	20	74.02, 76.74
BOLTBE	Thepouchplug Group [20]	8, 12, 16	29.50, 32.50, 31.50
Mantapp	Mantapp	2, 4	32.43
EGP	EGP [19]	6, 9, 14, 20, 24	33.00, 34.00, 36.00
YOMIWISE	No results found	4, 10, 14	62.70
LYFT	British American Tobacco, BAT [9]	15, 15.5	78.00
ace.	Ministry of Snus [3]	10.5, 16	69.68
White Fox	GN Tobacco	16.5, 17.5, 19.5, 22.5, 34	73.58
ONZ	No results found	6	17.90
**SHOPEE**			
EGP	EGP [19]	2, 4, 6, 9, 24	33.00–36.00
VELO	British American Tobacco, BAT [7]	4, 4.5, 6, 10, 11, 14, 17	52.53, 53.14, 57.04, 57.62, 60.85, 61.54, 66.19, 74.53, 79.41, 133.01
YALO	No results found	9, 14, 20	61.73
NASTY NICPAX	NICPAX [2]	4, 6, 10	19.90
Bxd	No results found	3, 4, 6, 9, 10	37.99
The Club, GOAT, XO	Cobber Sweden [18], Consumer Brands International (CBI) [8], Sweden	9, 16	23.90
Jade Hare	Jade Hare Cooperate [14]	15, 25	24.76
Superouse	Superouse [17]	8, 10, 12, 14, 17, 20, 24	28.00–32.00, 28.00–35.00
Zyok	No results found	8, 10, 12, 14, 17, 20	27.10
iSOSWEE	No results found	2, 4, 10, 14, 20	22.00–35.00
ManTapp	Mantapp	2, 4, 6, 8	15.00–18.00
YOMIWISE	no results found	10, 14, 16	11.45–29.25
**GLOBAL STORES**			
NOMA	NOMA [15]	-	25.00
FREEZIE	FREEZIE [13]	6	19.90
**TikTok**			
Mantapp	Mantapp	2, 4	0.01
ONZ	No results found	6	0.01
NICPAX	NICPAX [2]	4, 6, 10	0.01
NOMA	NOMA [15]	-	-

RM: 100 Malaysian Ringgits about $US25.

### Online marketing on the two global sites

‘Noma’ was the first product. The price was 25 RM, but buying provided one free, on a purchase of four cans. The brand name ‘Noma’ is also associated with flavors, namely, mint, watermelon, and mango. Nicotine strength was not displayed, but messages of ‘power up with Noma’ possibly indicate a higher strength of nicotine. The product also had images of its use during sports and was placed on food plates and work desks to imply lifestyle-related marketing claims. Interestingly, ‘quit smoking on your terms’ was also displayed. The second product, ‘Freezie’, was available in various flavors; the nicotine strength was 6 mg in all flavors. ‘Freezie’ displayed that the product contains nicotine, a chemical that is addictive.

### e-stores on Lazada and Shopee e-commerce sites

*Product information*

The 35 e-stores identified on e-commerce sites had different brand names such as ‘Velo’, ‘Zyn’, ‘Boltbe’, ‘77’, ‘Mantapp’, etc. The manufacturers were British American Tobacco, Philip Morris International, EGP, GN Tobacco, Swedish Match, etc. The prices of ONPs ranged 0.01–133.01 RM, and 34 (83%) of them had some product promotions such as discounts, buy one free, and free delivery (Table 1). All the e-stores displayed information in both Chinese and English about an array of flavors, of which the most common were some ‘mint’, ‘berry’, ‘mango’, ‘watermelon’, and ‘grape’ (Supplementary file Appendix B). There were 263 different flavors, and they were usually named using terms such as ‘Nordic spirit, ice cool, slim’. About a third of the e-stores contained written and/or pictorial instructions (14; 34%) on the use of the ONPs. These e-stores provided step-by-step instructions of use, supported by pictures to place them between the upper gums and buccal mucosa (Table 2).

**Table 2:** Descriptive statistics of regulatory language used, product-related information/content, and marketing-related slogans, strategies, and claims about ONP products on 41 e-stores in Malaysia during September–November 2025

*Information*	*n (%)*	*Marketing claims*	*n (%)*	*Prices and promotions*	*n (%)*	*Regulatory*	*n (%)*
Nicotine disclosure	21 (51)	Health-related claims	10 (24)	Prices range	RM 0.01–133.01	Age verification	0 (0)
Display of nicotine strength	39 (95)	Smoking cessation claims	20 (49)	Promotions	34 (83)	Identification proof	0 (0)
Tobacco-free	17 (41)	Reduced secondhand smoke	11 (27)	Flavors	41 (100)	Age warning	2 (5)
Nicotine is addictive	2 (5)	Convenient to use Product appeal	19 (46) 23 (56)	Instructions to use	14 (34)	Health warning	3 (7)

RM: 100 Malaysian Ringgits about $US25.

*Regulatory information*

The presence of nicotine disclosures, i.e. the term ‘nicotine’ or a statement such as ‘this product contains chemical nicotine’, was stated in an e-store or on the product in 21 (51%) e-stores. However, only 2 (5%) e-stores stated that nicotine is addictive (Table 3). In addition, more than a third (17; 41%) of the e-stores stated that the product is ‘tobacco-free’ using various phrases such as ‘100% Tobacco FREE, NO tobacco, zero tobacco, tobacco-free, snus-free’, etc. Notably, most (95%) of the stores displayed several strengths of nicotine in milligrams (mg), and these ranged from 2 to 50 mg (Table 1). Age verification and/or any photo identification document proof to verify the age of the potential buyer was not required in any of the e-stores. However, two e-stores had warnings against use by the underage (aged <18 years), three had health warnings such as an upset stomach due to spiciness, not recommended for use during pregnancy, and non-smokers (Table 2; and Supplementary file Appendix A).

*Marketing claims*

The most common marketing claims made in the stores were product appeal (23; 56%) and easy and convenient to use (19; 46%). Messages on e-stores described taste, aroma, energy, flavorful smoothness, refreshing, etc., to increase the appeal of ONPs. Commonly used quotes such as ‘enjoy everywhere’, ‘anywhere, anytime’, and ‘convenient and easy to use’ were present in the stores to attract customers. Smoking cessation claims were present in half the e-stores (20; 49%) (Table 3). Messages included ‘relief from addiction and nicotine cravings’, ‘relieve addiction instantly’, ‘stop smoking today’, ‘quick absorption’, and ‘long release to assist in quitting’ (Table 3). In 11 (27%) e-store claims on reduced secondhand smoke, such as ‘clean and environmentally friendly’, ‘NO smoke, smell or embarrassment’, ‘advantages of SNUS - does not generate the smoke like a cigarette’, etc., were present. Health-related claims were present for 10 (24%) of the e-stores. Messages such as ‘stop bad breath and teeth sensitivity’, ‘99% less toxicants than a cigarette’, ‘without the harmful effects of tobacco’, ‘will not stain teeth’ were present (Table 2).

## DISCUSSION

Our study showed that online marketing was mainly present on e-commerce websites in Malaysia. The ONP brands were ‘Velo’, ‘Zyn’, ‘EGP’, etc. The content analysis revealed that ONPs were being promoted by offering discounts, a wide array of flavors, and marketing claims related to being tobacco-free, convenience to use anywhere/everywhere, and overcoming cravings to smoke and quitting cigarette smoking. Most of the e-stores displayed wide-ranging strengths of nicotine, but e-stores seldom explicitly stated the presence of nicotine and the potential of nicotine addiction, provided health warnings; they did not have age verifications or warnings against use by minors.

The market size of the ONPs has been increasing^24^, and our study shows that ONPs have expanded to newer markets such as Malaysia. Advertising and marketing claims about ONPs on various media, including direct mail, have been reported in England^15^, and the United States^16-18^, but literature about marketing and sales of ONP from LMICs is lacking. Multichannel advertisements on social media (TikTok and Instagram), and public spaces that contained visually appealing content, influencer partnerships, and event sponsorships were reported from England^15^. Product promotion via discounts (up to 99%) and free deliveries is reported for ONPs^16^ and e-cigarettes^21^. Though Malaysian social media advertisements on ONPs and the presence of retail stores were limited, and most marketing and sales were occurring via e-commerce sites, links to join ONP discussion groups on social media and offers to ship ONPs to Malaysian online retailers outside Malaysia, highlight the need to implement a cross-border ban on tobacco advertisements and sponsorships, including the sales^25^. E-stores contained a wider range of ONP brands than commonly reported brands, such as ‘Zyn’, ‘Onz’, ‘Velo’, ‘Nordic Spirit’, and ‘Rogue’, in previous studies^15-18^. Notably, much of the e-store content was in dual language, namely English and Chinese, including the instructions to use and names of the flavors, suggesting a Chinese origin.

Most e-stores did not show warnings against minor use, though a few stated they are for adults only and instructed to keep ONPs out of children’s reach and warned about use during pregnancy. Interestingly, just one e-store mentioned that ONPs were not for non-smokers. Even e-cigarette-related content on online retail stores^21^ and social media, seldom contains age and/or health warnings^26^. Though the presence of nicotine is implicit, only half of the e-stores explicitly stated the presence of nicotine in product-related information, or very few placed disclaimers that nicotine is addictive. Our previous study of online retailers also found very few e-cigarette retailers that placed nicotine disclaimers^21^. Earlier studies about ONPs did not report the nicotine content^16-18^. The strengths of nicotine in ONPs ranged from 2 to 50 mg, much higher than the 1–4 mg recommended in lozenges, gums, and tablets that are recommended for smoking cessation^27^. The strengths displayed did not specify the amount of nicotine present per pouch in most stores. Despite the lack of nicotine disclaimers, the broad range of nicotine strengths suggests marketing of ONPs to those who are attempting to quit smoking, choosing higher strengths to overcome cravings, and to those who are nicotine-naïve customers, who can choose lower strengths. Though most e-stores displayed various flavors, the full contents of the products were seldom disclosed. An array of flavored ONPs was also reported from the United States^16,17^, known to increase the appeal of the products to adolescents, young adults, and novice users of tobacco products^28^.

The e-stores that sold ONPs contained mostly marketing claims related to health and the convenience of use. The main text presented the health-related claim as ‘Tobacco-free’. However, it has been reported that nicotine is present in ONPs as either synthetic or tobacco-derived^3^. Additionally, there were also claims related to no secondhand smoke, environment-friendly, and smoking cessation. In three studies from the United States, similar marketing claims have been identified from ONP advertisements via various media^16-18^. The studies from the United States mainly made marketing claims related to the convenience of ONP use in multiple contexts, places, tobacco-free, smoke-free, vape-free, spit-free, etc., but no marketing claims related to smoking cessation. Still, health-related and alternative tobacco claims were reported in studies from the United States^17,18^. About half of the e-stores made smoking cessation claims such as ‘stop smoking today’, ‘relief from addictions and craving’, ‘helpful to quit’, etc. The messages related to tobacco and snus free, no secondhand smoke (SHS), and environmentally friendly, in addition to being convenient to use anywhere and everywhere. Business-to-business advertisements have made similar marketing tactics^29^. ONP manufacturers have used social media such as TikTok to portray ONPs for recreational purposes, targeting the youth^30^ rather than harm reduction or smoking cessation, on exclusive use of ONP^31^. Though a few e-stores were marketing ONPs with claims of being a smoking cessation aid, evidence of the same is currently lacking^10^. Marketing claims of convenience of use seem to encourage maintaining nicotine use in places where perhaps smoking and vaping are prohibited.

On the e-stores, most of the marketing claims were related to health. In the absence of any evidence of the possible health effects of ONPs relative to cigarette smoking, the manufacturers seem to be using this as a marketing strategy. However, evidence of possible health effects, particularly on oral health, is emerging^14,32^. Though the health-related claims were related to tobacco-free, snus-free, etc., it is noteworthy that some warnings related to possible gastric symptoms due to ‘spiciness’ and salivation were related to the user instructions. Few e-stores displayed warnings for non-smokers, pregnant women, and children. Emerging evidence about changes in oral mucosa and altered oral microbial flora^33^ may raise questions about health-related marketing claims about ONP, yet ONPs are lower harm products than traditional cigarettes. The nicotine present in ONPs has been shown to contain higher freebase nicotine levels than protonated nicotine, which may lead to higher levels of addiction and cardiovascular effects^34^ than the traditional snus^35^.

### Limitations

This content analysis was done on the e-stores that sold ONPs. Though our search did not identify other advertisements or retail stores, we cannot rule out other channels via which ONPs may have been marketed and sold. ONPs are rapidly evolving product categories, the information about marketing and sales information available on the e-stores are only reflective of the period September to November 2025. We did not aim to analyze all brands of ONPs. The results of our study do not reflect those of any specific brands sold on the e-stores. Therefore, we could not compare the marketing claims according to the brands. The coding was done by two independent coders and verified by the third coder, and the codebook was adapted from similar research on e-cigarettes^21^, yet we cannot rule out missing some codes or misclassification of codes.

### Implications for research and regulatory policy

Further research is needed to monitor ONP advertisements, marketing, and sales via various channels to monitor how the manufacturer’s strategies evolve in the Malaysian context. It is also important to measure the impact of online marketing and sales via e-stores, subsequent use of ONPs^6^ and user characteristics^7^. The absence of age verifications and higher strengths of nicotine and flavors are all suggestive of marketing strategies to entice the youth into nicotine addiction. Reported literature on ONP regulations classifies Malaysia as a country where ONPs are not in the market and are regulated by classifying nicotine as a poison^8^. Stricter implementation of Act 852 by covering ONPs is needed to regulate them to prevent youth and nicotine-naïve individuals.

## CONCLUSIONS

Our study provides evidence for ONP marketing and sales in Malaysia. E-stores were marketing ONPs of varying strengths of nicotine, and myriad flavors were promoted by offering discounts and had claims related to product appeal, convenience of use, and smoking cessation. Surveillance of e-commerce and other media is necessary to monitor the marketing tactics of manufacturers and inform about potential regulations on ONPs.

## Supplementary Material



## Data Availability

The data supporting this research can be found in the Supplementary file.
