# Supplementary material for: Marketing of oral nicotine pouches on Malaysian social media and e-commerce websites
Source: Tob Induc Dis. 2026 May 29;24:10.18332/tid/219213. doi: 10.18332/tid/219213 (PMC13366215; doi:10.18332/tid/219213)
Supplement: Supplementary file 1 [file TID-24-77-s1.pdf]

Appendix A

The list of codes used, their definition, and textual examples (visuals are shown in Appendix C) used in coding of ONP products available on 41 e-stores in Malaysia during September- November 2025

Tobacco-/nicotine- related	Definition	Example text
Nicotine disclosure	Conveys that the product contains nicotine	• This product contains tobacco-free nicotine.<br>• Warning: This product contains nicotine,<br>• "Enjoy the slow release of nicotine from lip smoke", "Nicotine Pouches''
Nicotine is addictive	Statements related to the addiction to nicotine	• Nicotine is an addictive chemical<br>• Smokeless tobacco is addictive.
Tobacco-free	Conveys that the product does not contain tobacco products like cigarettes or traditional snuff	• 100% Tobacco FREE,<br>• NO tobacco<br>• zero tobacco<br>• Tobacco-free<br>• snus free
Display of strength	The amount of nicotine present in the pouch is displayed on the e-store or the product	2 mg, 4 mg, 8 mg etc.
Marketing Claims		
Health-related claims	Conveys the health benefits of using the product	• stop bad breath and teeth Sensitivity",<br>• "Will not stain teeth,<br>• "A healthier lifestyle experience",<br>• ONZ delivers all the satisfaction, minus the drawbacks of traditional smoking methods<br>• 99% less toxicants than a cigarette"<br>• without the harmful effects of tobacco.
Smoking cessation claims	Conveys that the product will assist those who smoke to	• Relieve addiction instantly,

	quit	• Stop smoking today<br>• Relief from nicotine cravings, provides relief from nicotine cravings,<br>• Want to quit smoking? This is the easiest way<br>• Quit Smoking on your terms
Reduced secondhand smoke	Conveys that others are not exposed to the smoke emitted from cigarettes	• clean and environmentally friendly,<br>• "Smoke Free"<br>• Advantages of SNUS - does not generate smoke like a cigarette<br>• no smoke, smell, or embarrassment<br>• Smoke-free experience<br>• smokeless and not disturbing
Convenient to use anywhere or to circumvent smoke-free policies	Conveys that can be used in places where smoking is prohibited	• enjoy everywhere, “anywhere, anytime, “<br>• convenient and easy to use<br>• small and easily portable<br>• Use it discreetly, anytime, anywhere (in meetings, on planes, or in the cinema)<br>• It can be used in smoking-prohibited places,<br>• no burn”,<br>• Use it discreetly, anytime, anywhere (in meetings, on planes, or in the cinema). Can be used in smoking-prohibited places<br>• They offer a cleaner, convenient way to satisfy cravings anytime, anywhere
Product appeal	Conveys messages that enhance the attractiveness of the product to entice potential purchase	• Energy booster and a Flavorful experience<br>• Natural Coffein - Vitamin B5 - Sugar-free”,<br>• Unlike gum or sprays, it tastes good,<br>• Taste Profile: Fresh",

		• smooth, mess-free, and flavorful experience, ONZ delivers all the satisfaction<br>• Superb Aroma<br>• provide quick and refreshing nicotine satisfaction orally<br>• moist pouches<br>• designed for rapid absorption, long-lasting"<br>• “Main ingredients: alkaloids and other food-grade ingredients
Prices and Promotions		
Promotions		• Buy 2, get 3% off, buy 4, get 4% off,<br>• Spend RM50, 20%-50% off
Flavors	Displays available flavors	Strawberry, coffee, mint, etc.
Instructions to use	Provides instruction on how to use by text or pictures	• pictorial instruction or text, such as<br>• no need to chew or swallow, and the effect will last from 30 minutes to one hour<br>• putting on the upper lip will prevent excessive production of saliva.<br>• dispose of the used pouch responsibly
Regulatory		
Age verification	Age verification that restricts minors from accessing the product on the e-store	Confirmation of age by click-through option: “Are you above 18 years old? Yes/ No.
Identification proof	Requirement for age verification by providing an identification document	Please provide an identification number such as a national identification card, driver’s license, a student ID or other proof of age.
Age warning	Conveys messages to prohibit use by minors,	• Only for use by adults.<br>• This product is for adults aged 18+ and over only, please keep out of reach of children

Health warning	Cautions about possible harms from the product	• It tastes spicy and will cause stomach upset if mixed with saliva<br>• Do not use this product if you have severe heart disease, are pregnant, or breastfeeding.<br>• Not to be used by non-smokers, please consult your health care professional before use.

Appendix B

List of flavors found on each store by brand names of ONPs available on the e-stores during September- November 2025

**Brand**	Flavors
VELO	V Mighty Peppermint Max, V Ice Cool Strong-Cool Mint, V Ice Cool Strong (EU)-Cool Mint European version, V Ruby Berry Strong (EU)-Akihito European, V Blushy Berry Akiko Berry, V Ruby Berry Mini (EU)-Mini Akiko Berry European Edition, V Ice Cool Zero-Cool Mint Zero, V Tropic Breeze Zero-Tropical Fruit Zero, V Ice Cool Storm ZERO Storm Mint Zero, V Twisted Pineapple J Caribbean Fruity, V Lime Flame-Mexican Pepper New Edition, V Lofty Liquorice-Licorice Aromatherapy New, V Frozen Cloudberry, V MINI GROOVY GRAPE-Little Emperor Purple Grape, V Punchy Lime-Fresh Sweet Lime, V tangled berry-raspberry essence, V Cinnamon Flame Cinnamon Sweet, L Hot Berries-Passion Berry, V MINI SPIFFY SPEARMINT-Light Mint, V Eucalyptus X strong, V Mango Flame-Mango Flame, V Humble Spearmint Mini, V Elderflower Spritz-Elderflower fragrance, V Nutty Virginia Mini-Nuts Tobacco, V Royal Purple Slim Strong-Big King Purple Grape, L Cucumber Mint Strong-Cucumber Mint, V Ice Cool Mini Champion Mint, V ORANGE SPARK Citrus Sweet Rhyme, L Citrus Mint Mini Citrus Fruity, L Eucalyptus & Honey Regular, L Eucalyptus & Honey Strong Honey Eucalyptus, V PEPPERMINT STORM Storm Mint, V Mighty Peppermint Ultra Large Mint, L Cool Eucalyptus X Strong, L Pure Mint Mini Pure Mint, L LYFT Cool Air X Strong Oversized Mint, V ARCTIC GRAPEFRUIT Frost Grapefruit, V Wintery Watermelon Mini Iced Watermelon, V Icy Berries Cool Berry, V Mighty Peppermint, L Black Currant Slim Strong Blackcurrant Incense Expired, V Witty Spearmint Menthol, VELO MAX Mighty Peppermint Slim Fresh Mint – Menthol Peppermint, VELO Frozen Cloudberry Slim All White cloudberry
ZYN	Black Cherry Mini Dry- Almond Cherry, Cool Mint Cold Mint
SIBERIA	Semi-red mint (mint), All Red Mint (mint), Semi-brown leather (leather), Full brown leather (leather), Half Black Red Original (original), All Black Red Original (original), Semi Black Original (original), All Black Original (original), Half blue mint (mint), All Blue Mint, Half-red mintmini(mint), Semi Black Original mini(mint), White Lip Nicotine Pouches – Polar Mint Flavor(mint), White Lip Nicotine Pouches – Pure White Mint(mint), White Lip Nicotine Pouches – Red & White Mint(mint), White Lip Smoke White Bear Mint (mint), White lip smoke all white mint long (mint)
77	Ghost King Mint(mint), Icy Cola(cola), Cherry (cola), Vanilla Cola(cole), Fresh Mint(fresh mint), Sweet Strawberry (strawberry), Mixed Fruit(fruits), Raspberry (raspberry), Peach Mint(peachmint), Black currant, Ice mint, Icy Watermelon(watermelon), Classic Original(original), Mango Mint(tropical&mint), Apple Mint(apple&mint)
Göteborgs Rap É Lingon	Large White Huckleberries
VOLT	dark berries, mint citrus lime apple spearmint, mint (apple spearmint), (speamint) menthol (mint) mixed berry (strawberry), blueberry vanilla (blueberry) strawberry (berries), hawthorn Canned (hawthorn)
Oden's	Cold Extreme Slim White Dry Mint
Chainsaw	Menthol Mint
Lundgrens	Berry Tobacco

General	Bergamot Citrus
PABLO	Bubblegum bubble gum flavor
Kelly White	Fruity raspberry lemon
Vika	Juicy lemon fruity
Sting free	Strong blue mint
Röda Lacket	fruity tobacco
G.3	green tea (green tea), oak (oak)
Shunfeng	Mint fruit
BOLTBE	Tangy Orange, Passion Fruit, Citrus Mint, Ice Mint, Black Current
Mantapp	Mint, Harum-Mango Ice
EGP	Ice Watermelon, Mango, Refreshing Grape, Coffee, Ice Mint
YOMIWISE	Berry
LYFT	Fresh Eucalyptus Eucalyptus
ace.	Eucalyptus
White Fox	rose flavor (rose flavor), double mint (double mint), spearmint (spearmint), snow flint (mint), pepper mint (pepper mint)
ONZ	Fresh and Cool ICE Flavor
EGP	Ice Watermelon, Mango, Refreshing Grape, Coffee, Ruby Berry, Mixed Berries, Citrus Chill
VELO	Max Freeze, X-Freeze, Ice Cool, Polar Mint, Freeze, Liquorice, Caribbean Spirit, Spicy Pineapple, Cool Storm, Twisted Pineapple, Royal Purple, Frosty Grapefruit, Ruby Berry, Lime Flame, Orange Spark, Cinnamon Flame, Iced Melon, Nutty Virginia (Mini), Punchy Lime (Mini), Easy Mint (Mini), Citrus Mint (Mini), Royal Tea, Iced Melon (Mini), Royal Purple (Mini), Tropic Breeze, Strawberry, Energy Drink, Coffee, Mango, Ice Mint, Apple, Grapefruit Child
YALO	Mint, Watermelon, Grape, Mango, Fragrance, Red Cow, Holly, Frozen
NASTY NICPAX	Artic Mint, Blue Razz, Cherry Chase, Cool Mint, Lush Ice, Mango Ice, Mint Freeze, Pure Peach
bxd	Cool Mint, Watermelon, Mung Bean Smoothie, Cool Ice Point, Blueberry
The Club, GOAT, XO	Watermelon Chili, Spearmint, Ice Cool, Grape Ice, Blueberry, Tropical, Cool Mint, Blueberry 2, Wild Cherry, Coco Choco
Jade Hare	New Energy Drink, New Mint 25MG Old, New Coffee, New Tropical Fruit, New Mint 15MG, New Berry Cocktail, New Ice Coconut, New Ice Cream, New Crystal Grape, New Hand Lemon 11MG

Superouse	Lemon Ice 8mg, Litchi 8mg, Blueberry 10mg, Sweet Melon 10mg, Royal Purple 10mg, Ruby Berry 14mg, Max Freeze 17mg, Watermelon (8mg), Ice Spring (8mg), Grape Ice (10mg), Double Mint (12mg), Mango Ice (12mg), Pepper Mint (12mg), Apple Ice (14mg), Passion Fruit (14mg), Polar Mint (14mg), Max Ice Cool (20mg)
Zyok	Watermelon-8mg, Lychee-8mg, Grape-10mg, Mango Chill-12mg, Apple Chill-14mg, Ice Freeze-17mg, Ice Freeze-20mg
iSOSWEE	Watermelon 10MG, Blueberry 14MG, Coconut Ice 14MG, Green Apple 14MG, Ice Mint 14MG, Long Jing 14MG, Orange 14MG, Spearmint 14MG, Energy Drink 20MG, Spring Ice 20MG
ManTapp	Mint 2mg, Harum-Mango Ice 4mg, Choco Dino 4mg, Teh Limau 6mg, Watermelon Chill 8mg
YOMIWISE	A Hong Dragon, Blueberry Storm, Rose Blue, Ice Cream Citrus, Crystal Litchi, Watermelon Ice Cream, Ice Mineral Water, Polar Ice Mint, Succulent Mango, Raspberry Berry, Coconut Ice, Experience Loaded 4 A
NOMA	NOMA Freeze, NOMA Ice Cool, NOMA Mango, NOMA Watermelon
FREEZIE	Chilled Mint, Classic Mint, Citrus Ice, Espresso, Peppermint, Mango, Lemon Mint, Menthol, Spearmint, Orange

REFERENCES for manufacturer

1. *77 Nicotine Pouches UK Online | Havana House*. (n.d.). Retrieved November 7, 2024, from https://www.havanahouse.co.uk/product-category/tobacco-free-products/77-nicotine-pouches/
2. *About Us*. (n.d.). NICPAX. Retrieved November 19, 2024, from https://nicpouch.my/pages/about-us
3. *Ace*. (n.d.). Retrieved November 7, 2024, from https://snusdaddy.com/ace?srsltid=AfmBOopPUJKdyIbRvCSEBdQhhbB5pUtUZ6l_W_V69wL3aruM5NiHI9u3
4. *Brand: G.3 Snus*. (n.d.-a). Snusline. Retrieved November 7, 2024, from https://www.snusline.com/brand/g-3/
5. *Brand: Sting Free Nicotine Pouches*. (n.d.-b). Snusline. Retrieved November 7, 2024, from https://www.snusline.com/brand/sting-free/
6. *Brand: Vika Snus and Nicotine Pouches*. (n.d.-c). Snusline. Retrieved November 10, 2024, from https://www.snusline.com/brand/vika/
7. *British American Tobacco—Velo*. (n.d.). Retrieved November 7, 2024, from https://www.bat.com/brands-and-innovation/velo
8. *Buy GOAT Nicotine Pouches From SnusBoss*. (n.d.). Retrieved November 19, 2024, from https://snusboss.com/nicotine-pouches/goat/
9. *Buy LYFT Nicotine Pouches | Free delivery to EU & UK*. (n.d.). Retrieved November 7, 2024, from https://www.snusdirect.eu/lyft-nicotine-pouches
10. *Buy Pablo Nicotine Pouches: Variants and Prices Online*. (n.d.). Snusboss. Retrieved November 7, 2024, from https://snusboss.com/nicotine-pouches/pablo/

11. *Buy Siberia Nicotine Pouches | Order online -SnusDirect*. (n.d.). Retrieved November 7, 2024, from https://www.snusdirect.com/siberia-all-white-nicotine-pouches
12. *Buy VOLT nicotine pouches | Great prices & Fast shipping*. (n.d.). Retrieved November 7, 2024, from https://www.snusdirect.com/volt-nicotine-pouches
13. *Freezie Nicotine Pouches*. (n.d.). Freezie Malaysia. Retrieved November 19, 2024, from https://freeziemy.com/products/6mg
14. *JADEHARE TOBACCO-FREE NICOTINE POUCHES*. (n.d.). Retrieved November 20, 2024, from https://www.jadehare.net/
15. *Noma- a refreshing new nicotine experience anytime, anywhere.* (n.d.). NOMA. Retrieved November 19, 2024, from https://noma.my/
16. *Philip Morris International to invest over $800 million to meet growing Zyn market*. (n.d.). Retrieved November 7, 2024, from https://truthinitiative.org/research-resources/emerging-tobacco-products/philip-morris-international-invest-over-800-million
17. *superouse oral nicotine pouches manufacturer—Google Search*. (n.d.). Retrieved November 20, 2024, from https://www.google.com/search?q=superouse+oral+nicotine+pouches+manufacturer&oq=superouse+oral+nicotine+pouches+man&gs_lcrp=EgZjaHJvbWUqBwgBECEYoAEyBggAEEUYOTIHCAEQIRigATIHCAIQIRigAdIBCTExMzU5ajBqN6gCALACAA&sourceid=chrome&ie=UTF-8
18. *The Club Nicotine Pouches—SnusMe.com*. (n.d.). Retrieved November 19, 2024, from https://snusme.com/the-club-nicotine-pouches.html
19. *What EGP - EGP*. (2024, August 19). https://egpouch.com/what-egp/
20. *ZYN coolmint 3MG nicotine pouches private label manufacture oem* -. (2024, March 13). https://boltbe.com/product/zyn-coolmint-3mg-inspired-private-label-nicotine-pouches-services/

APPENDIX C

Screen shots of oral nicotine pouches sold on e-commerce websites (TikTok and global stores not included) during September- November 2025

LAZADA PRODUCTS

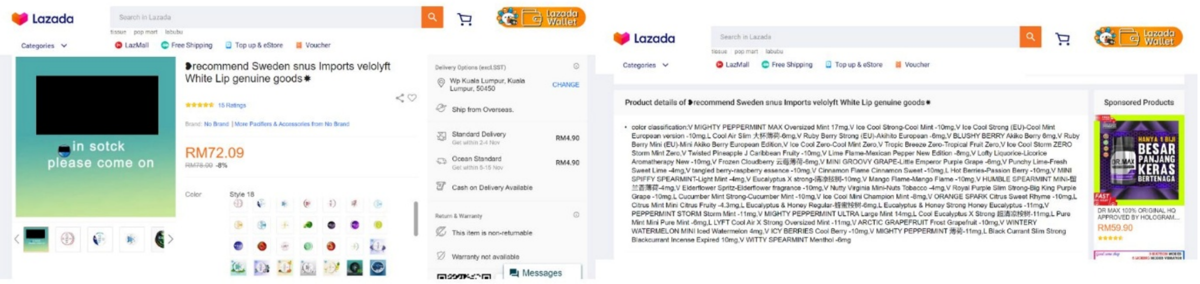

PRODUCT 1 – recommend Sweden snus Imports velolyft White Lip genuine goods

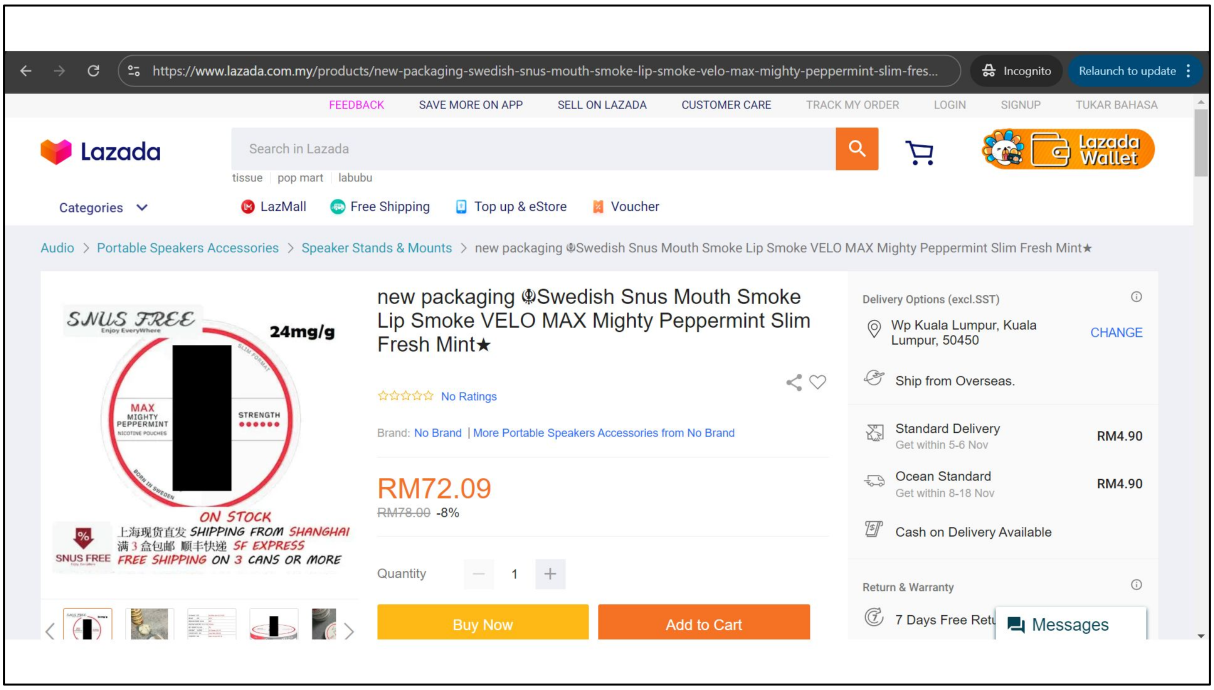

PRODUCT 3 - new packaging 🍭Swedish Snus Mouth Smoke Lip Smoke VELO MAX Mighty Peppermint Slim Fresh Mint

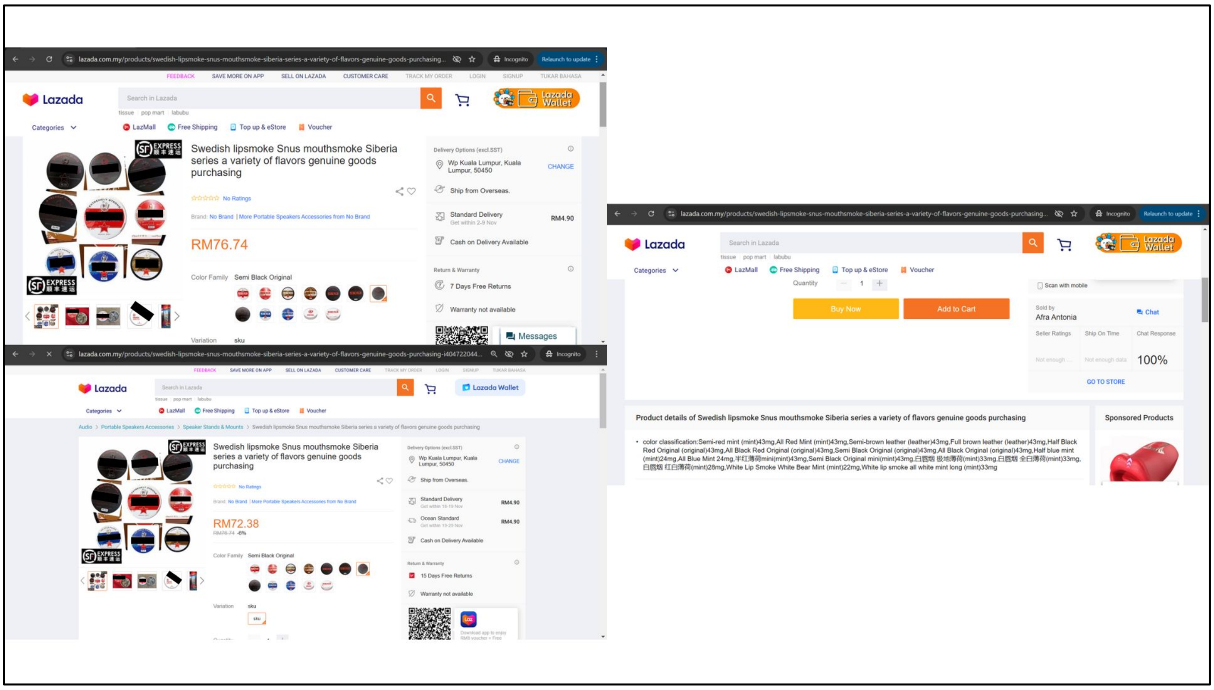

PRODUCT 4 - Swedish lipsmoke Snus mouthsmoke Siberia series a variety of flavors genuine goods purchasing

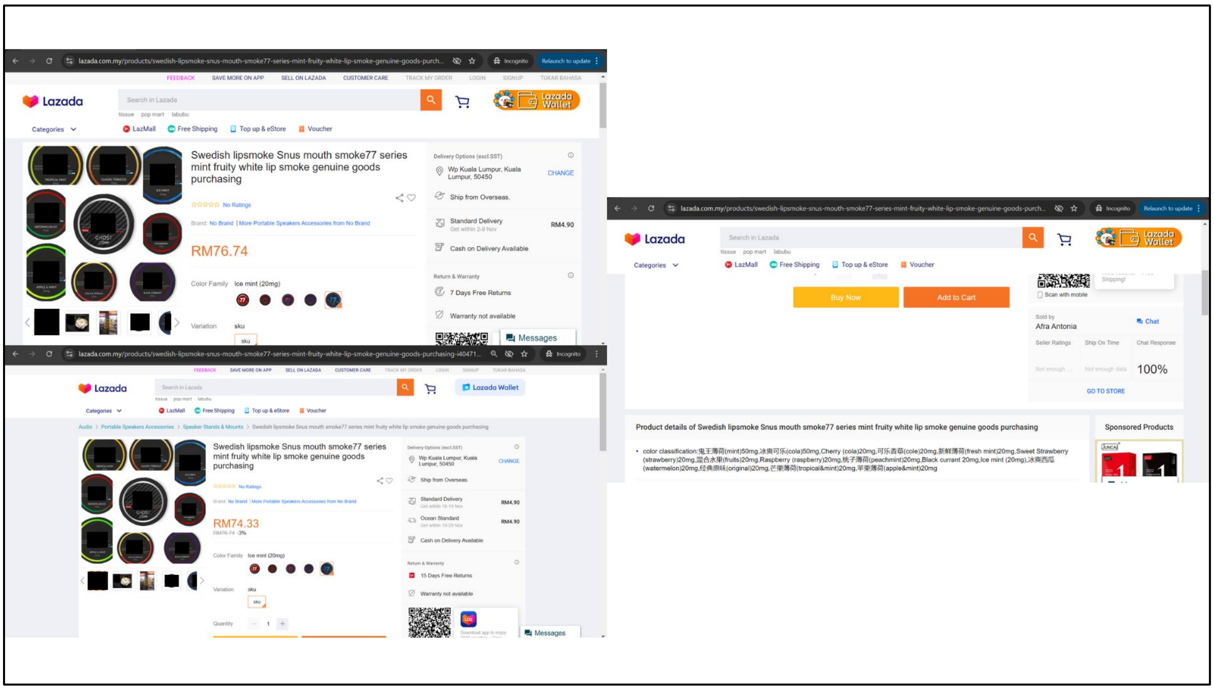

PRODUCT 5 - Swedish lipsmoke Snus mouth smoke77 series mint fruity white lip smoke genuine goods purchasing

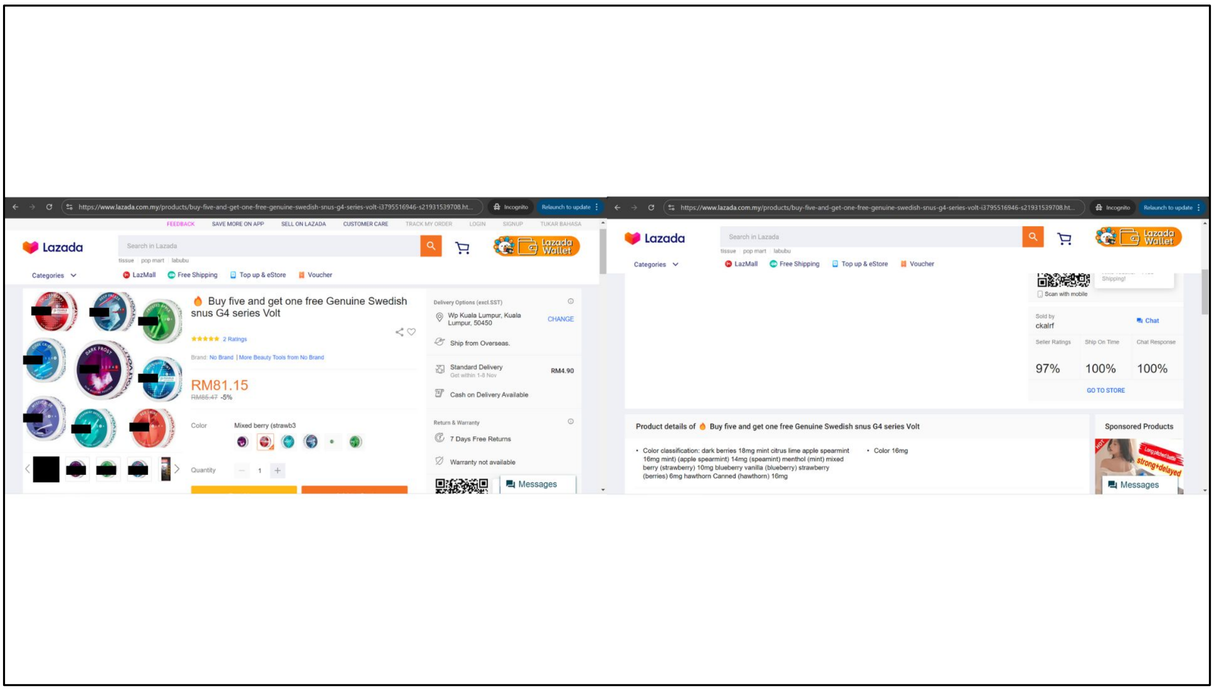

PRODUCT 7 - Buy five and get one free, Genuine Swedish snus G4 series Volt

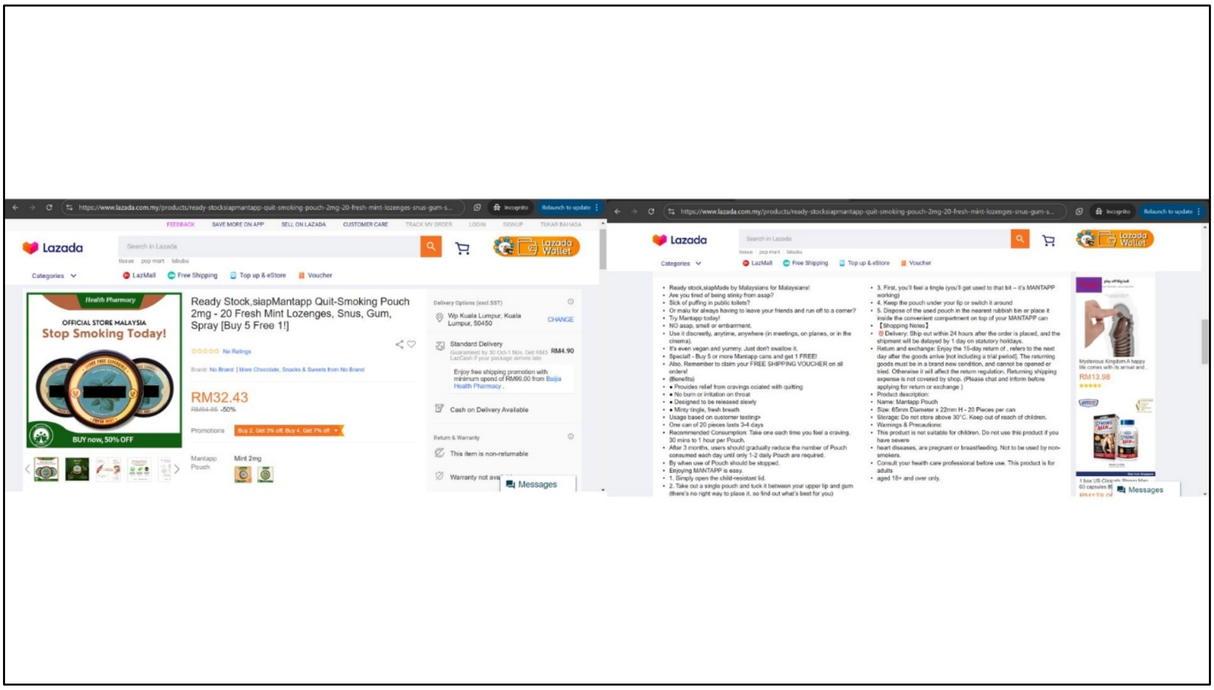

PRODUCT 21 - Mantapp Quit-Smoking Pouch 2mg - 20 Fresh Mint Lozenges, Snus, Gum, Spray

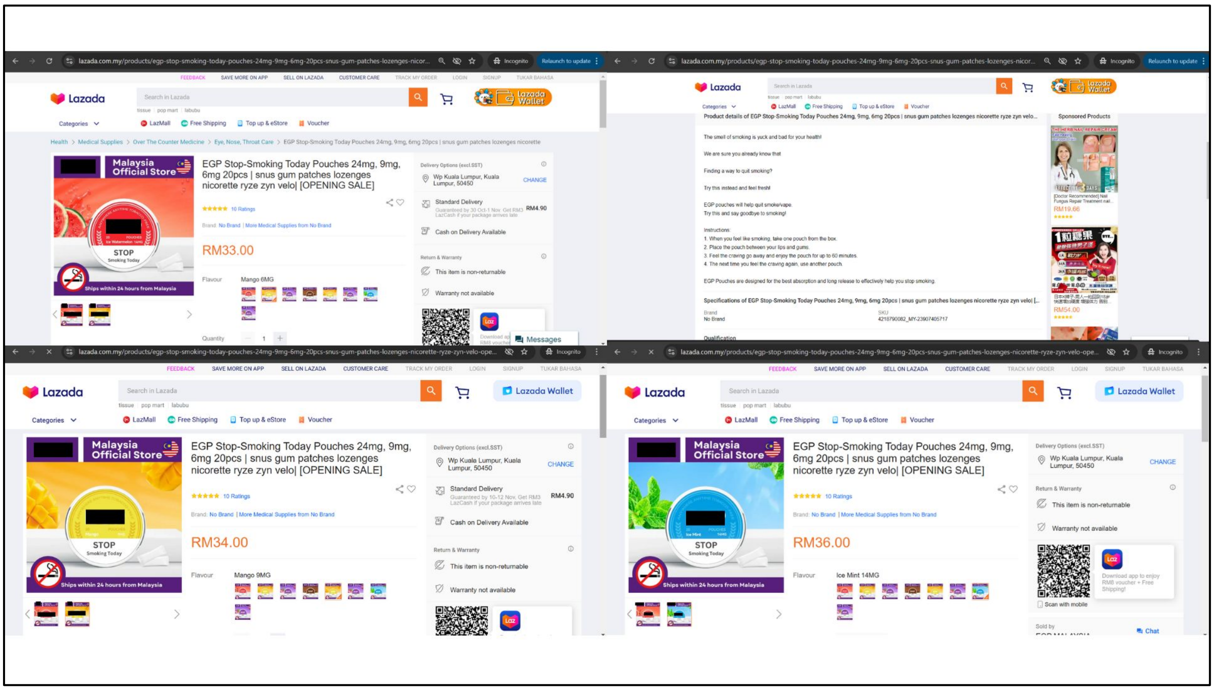

PRODUCT 22 - EGP Stop-Smoking Today Pouches 24mg, 9mg, 6mg 20pcs | snus gum patches lozenges nicorette ryze zyn velo

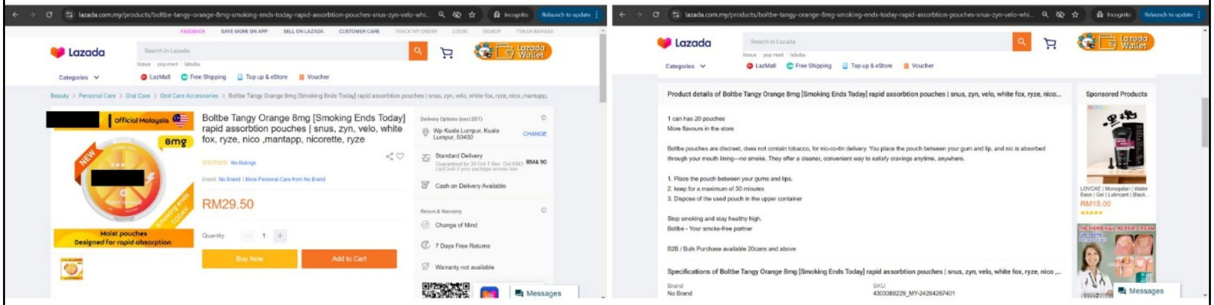

PRODUCT 23 - Boltbe Tangy Orange 8mg [Smoking Ends Today] rapid assorbtion pouches | snus, zyn, velo, white fox, ryze, nico ,mantapp, nicorette, ryze

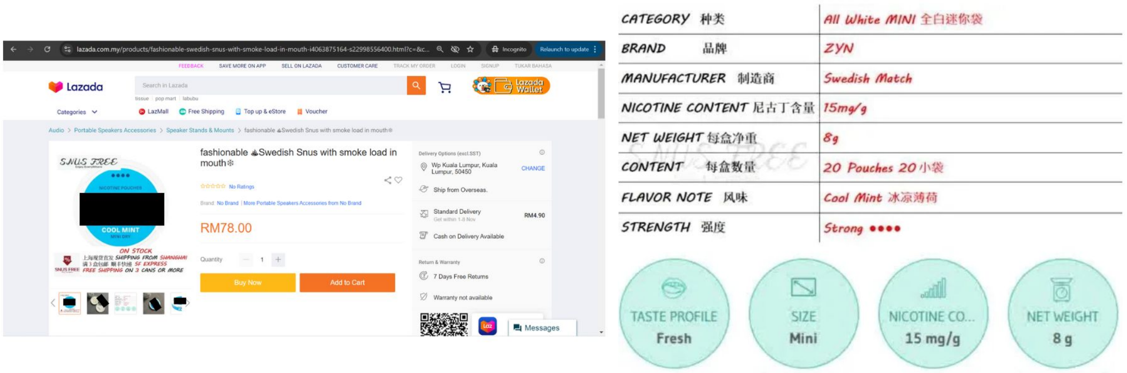

PRODUCT 24 - fashionable ♨Swedish Snus with smoke load in mouth

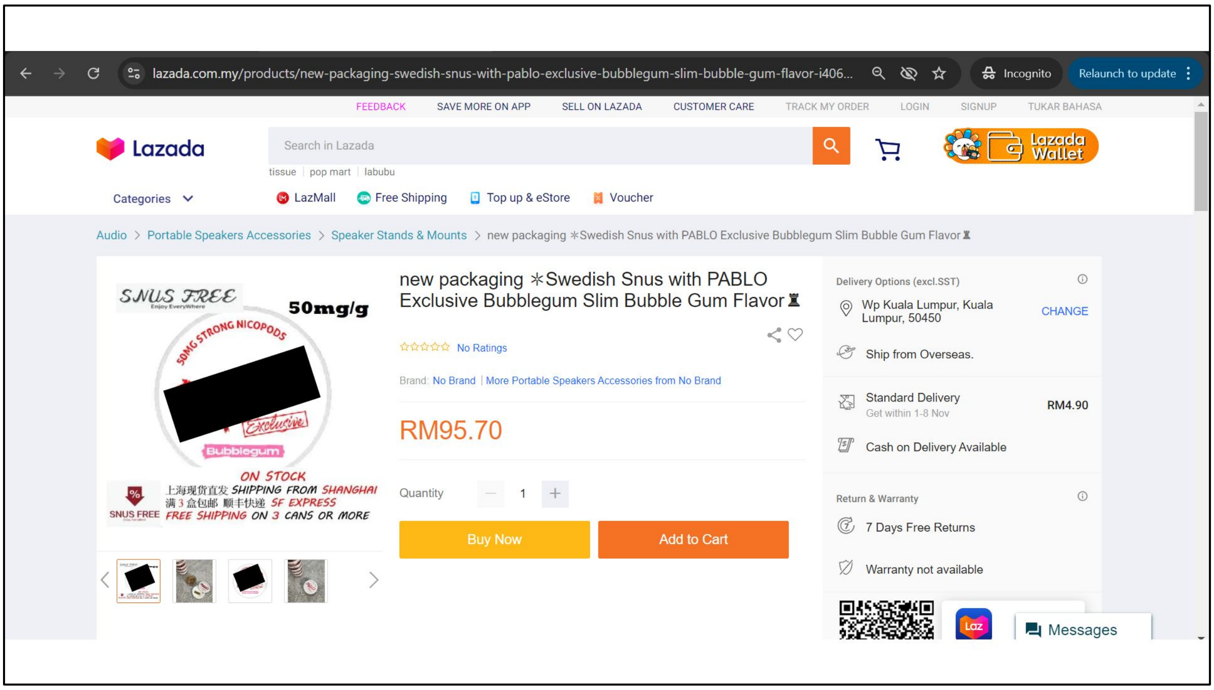

PRODUCT 25 - new packaging ✳Swedish Snus with PABLO Exclusive Bubblegum Slim Bubble Gum Flavor♜

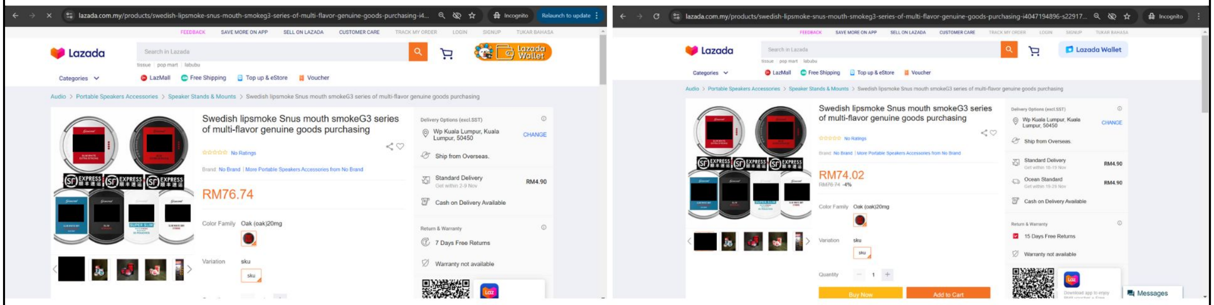

PRODUCT 26 - Swedish lipsmoke Snus mouth smokeG3 series of multi-flavor genuine goods purchasing

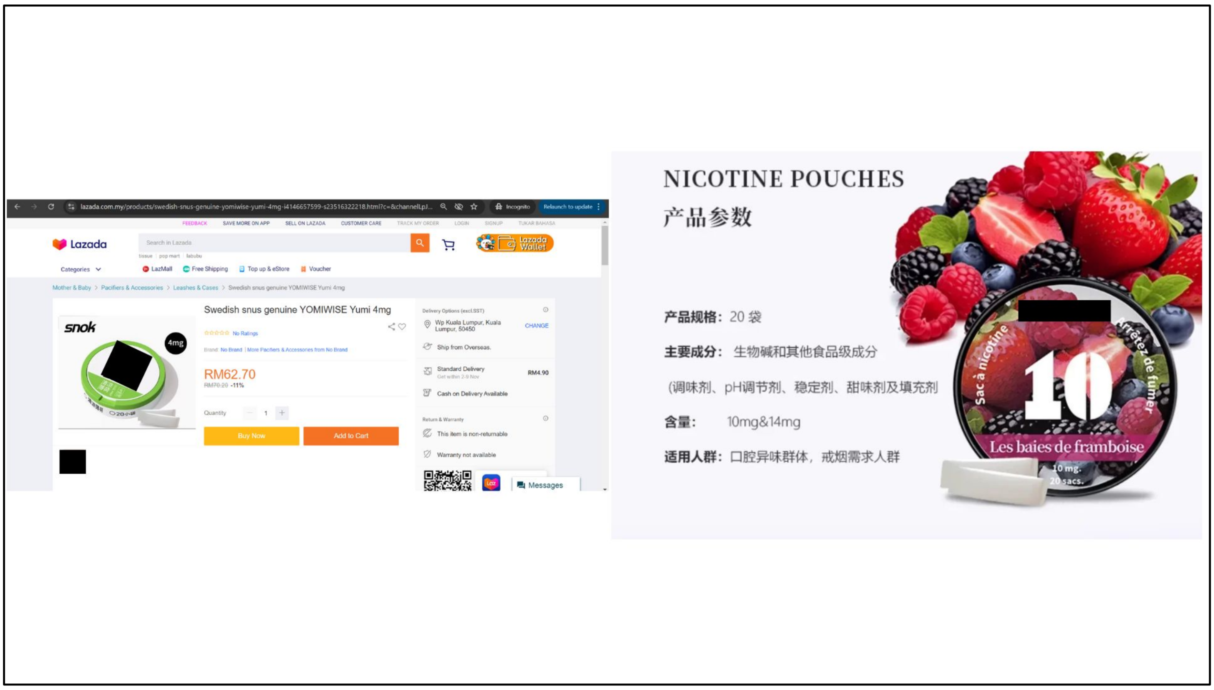

PRODUCT 27 - Swedish snus genuine YOMIWISE Yumi 4mg

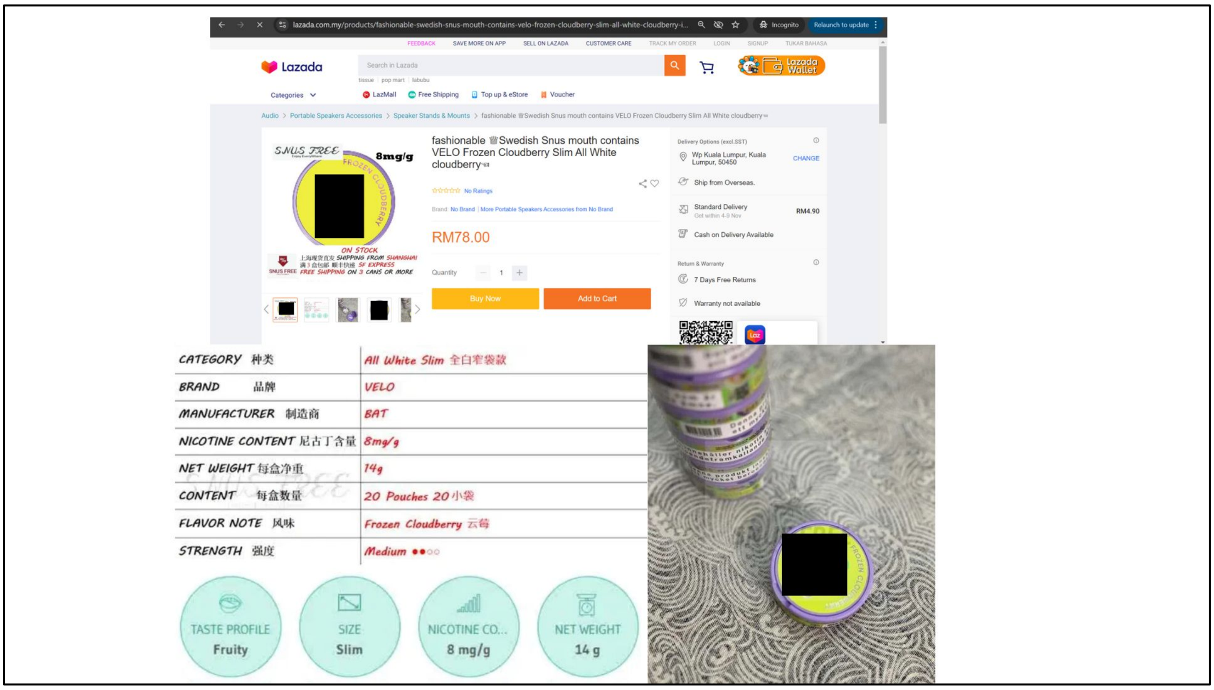

PRODUCT 28 - fashionable ♕Swedish Snus mouth contains VELO Frozen Cloudberry Slim All White cloudberry☜

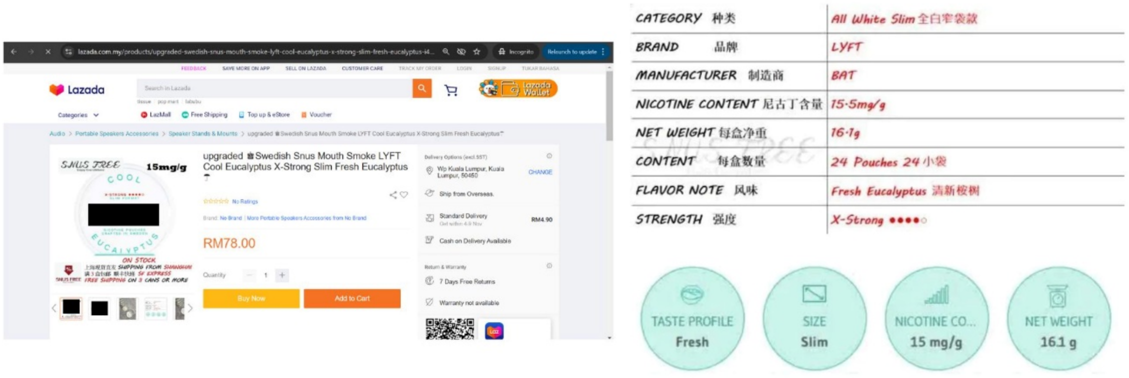

PRODUCT 29 - upgraded 🛍Swedish Snus Mouth Smoke LYFT Cool Eucalyptus X-Strong Slim Fresh Eucalyptus☂

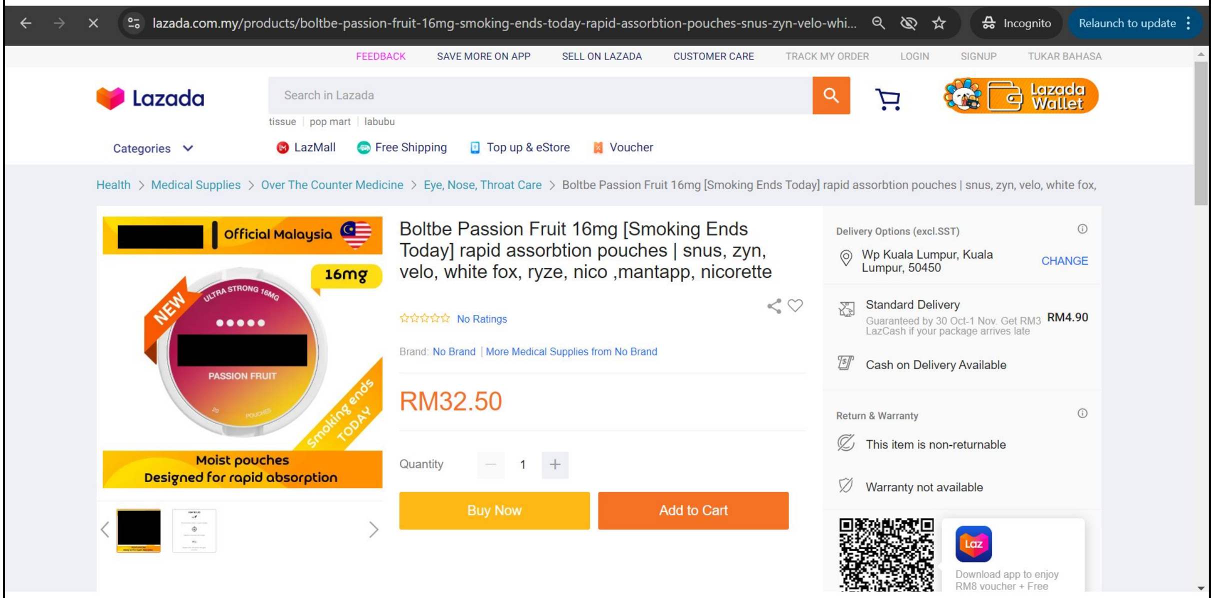

PRODUCT 30 - Boltbe Passion Fruit 16mg [Smoking Ends Today] rapid assorbtion pouches | snus, zyn, velo, white fox, ryze, nico ,mantapp, nicorette

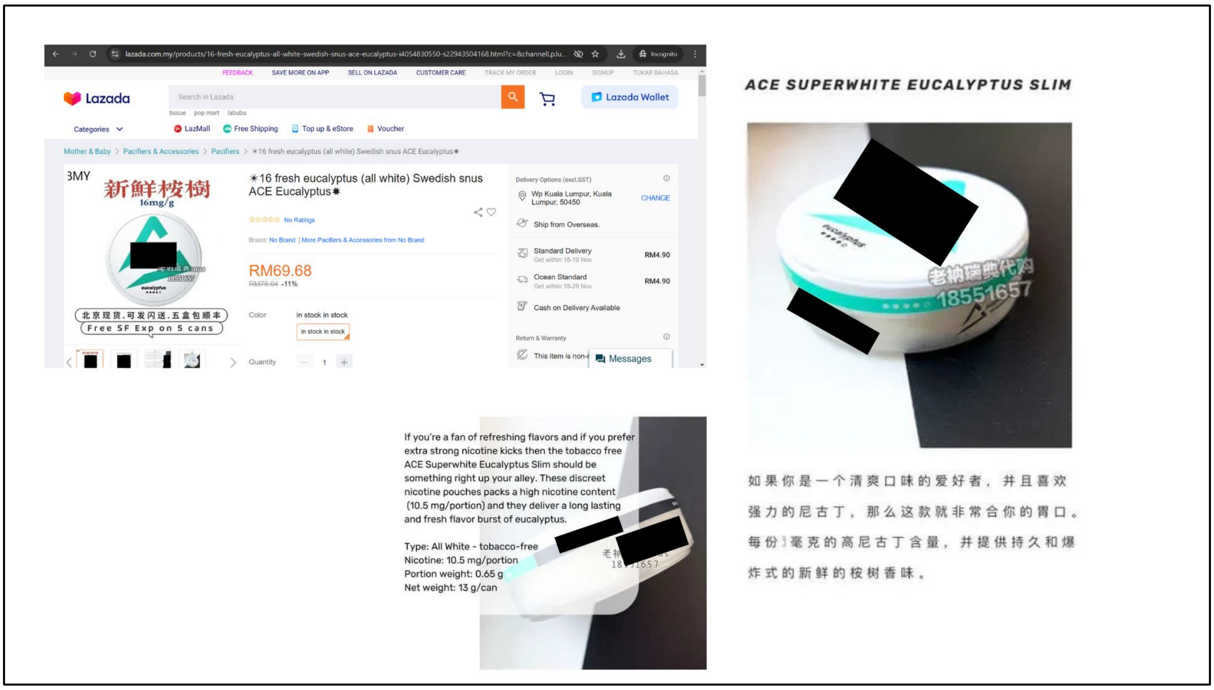

PRODUCT 31 - ✳16 fresh eucalyptus (all white) Swedish snus ACE Eucalyptus✸

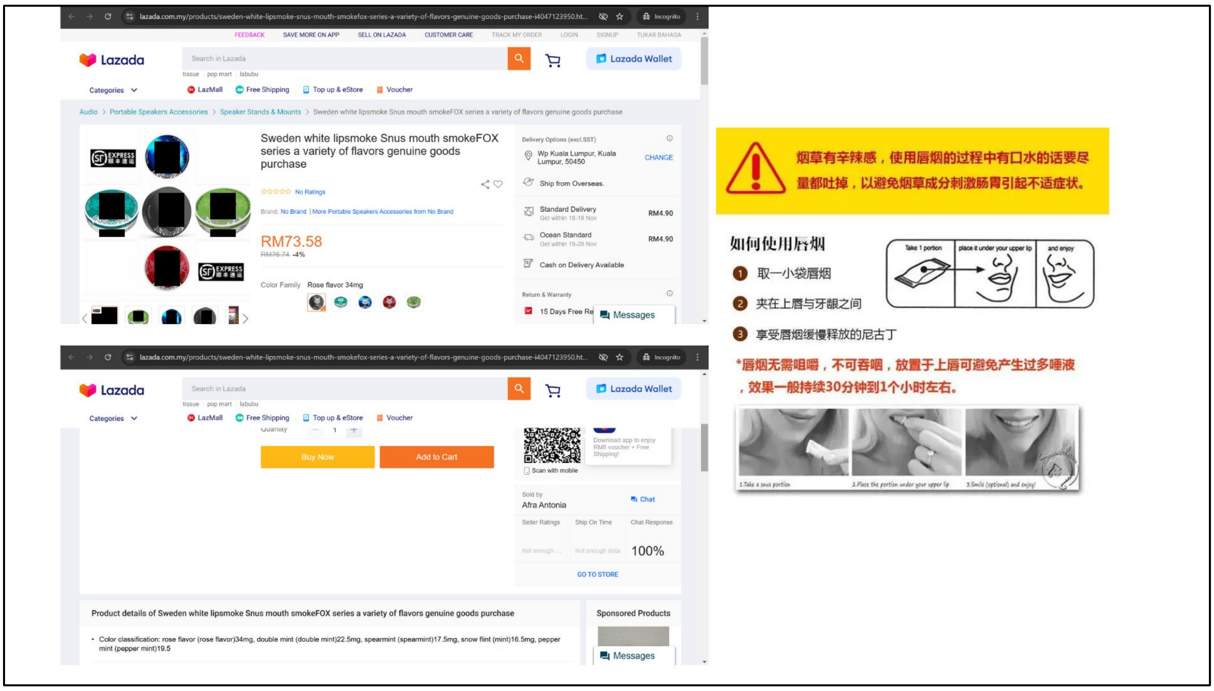

PRODUCT 32 - Sweden white lipsmoke Snus mouth smokeFOX series a variety of flavors genuine goods purchase

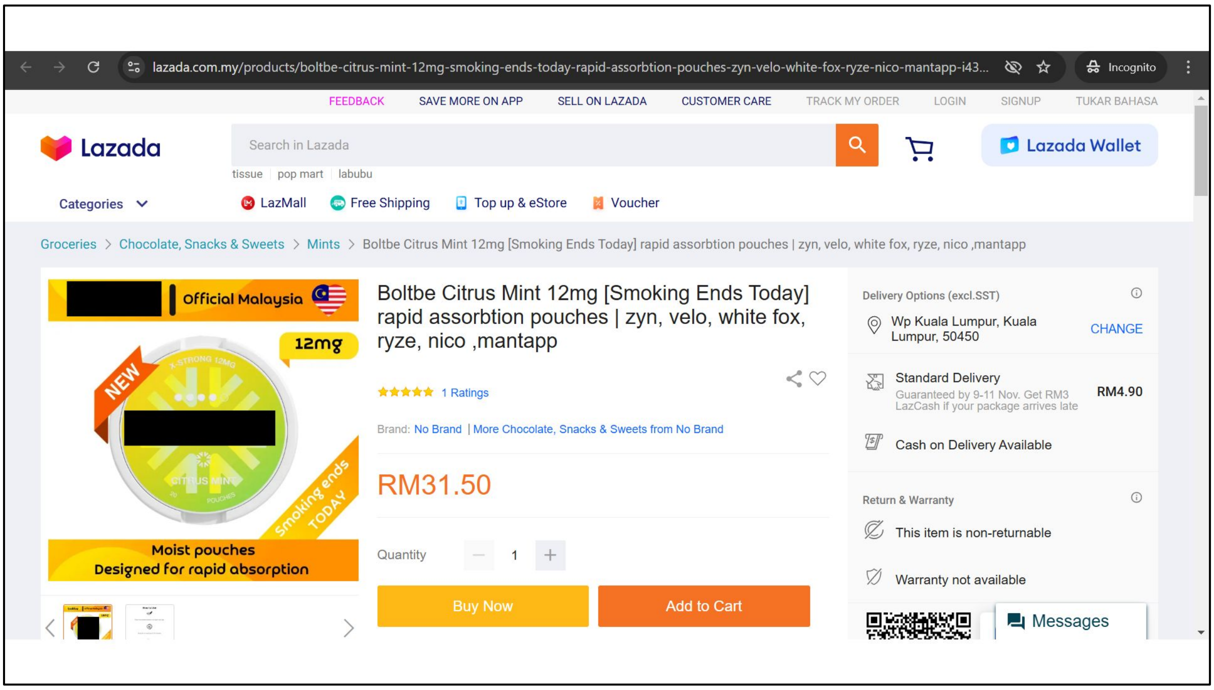

PRODUCT 33 - Boltbe Citrus Mint 12mg [Smoking Ends Today] rapid assorbtion pouches | zyn, velo, white fox, ryze, nico ,mantapp

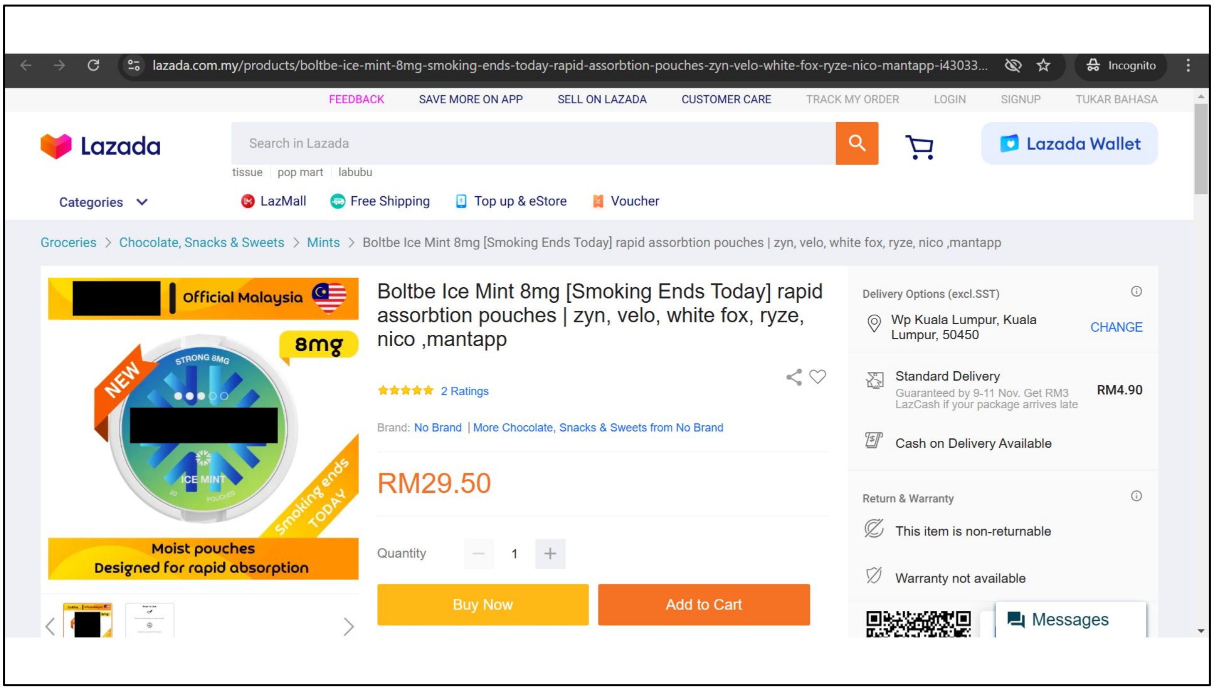

PRODUCT 34 - Boltbe Ice Mint 8mg [Smoking Ends Today] rapid assorbtion pouches | zyn, velo, white fox, ryze, nico ,mantapp

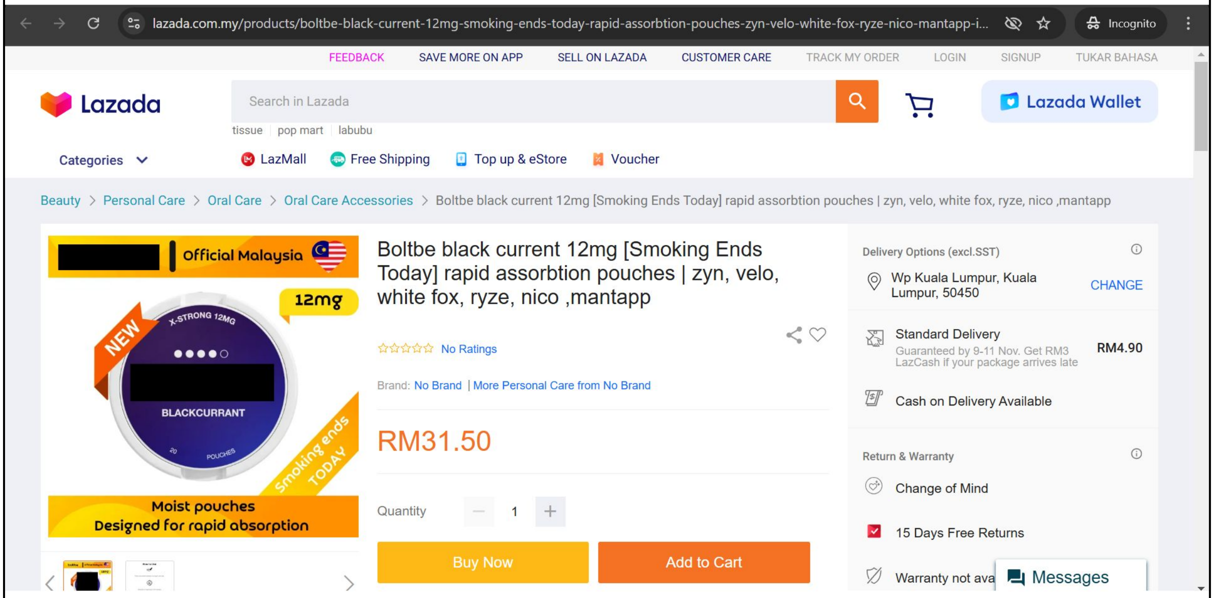

PRODUCT 35 - Boltbe black current 12mg [Smoking Ends Today] rapid assorbtion pouches | zyn, velo, white fox, ryze, nico ,mantapp

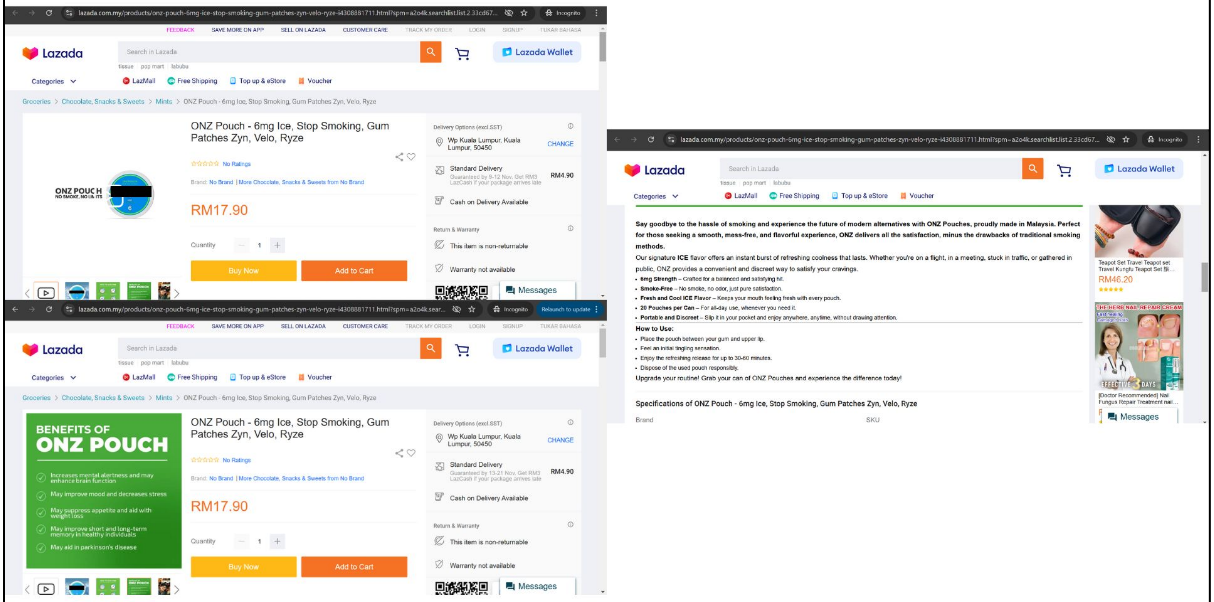

PRODUCT 36 - ONZ Pouch - 6mg Ice, Stop Smoking, Gum Patches Zyn, Velo, Ryze

SHOPEE PRODUCTS

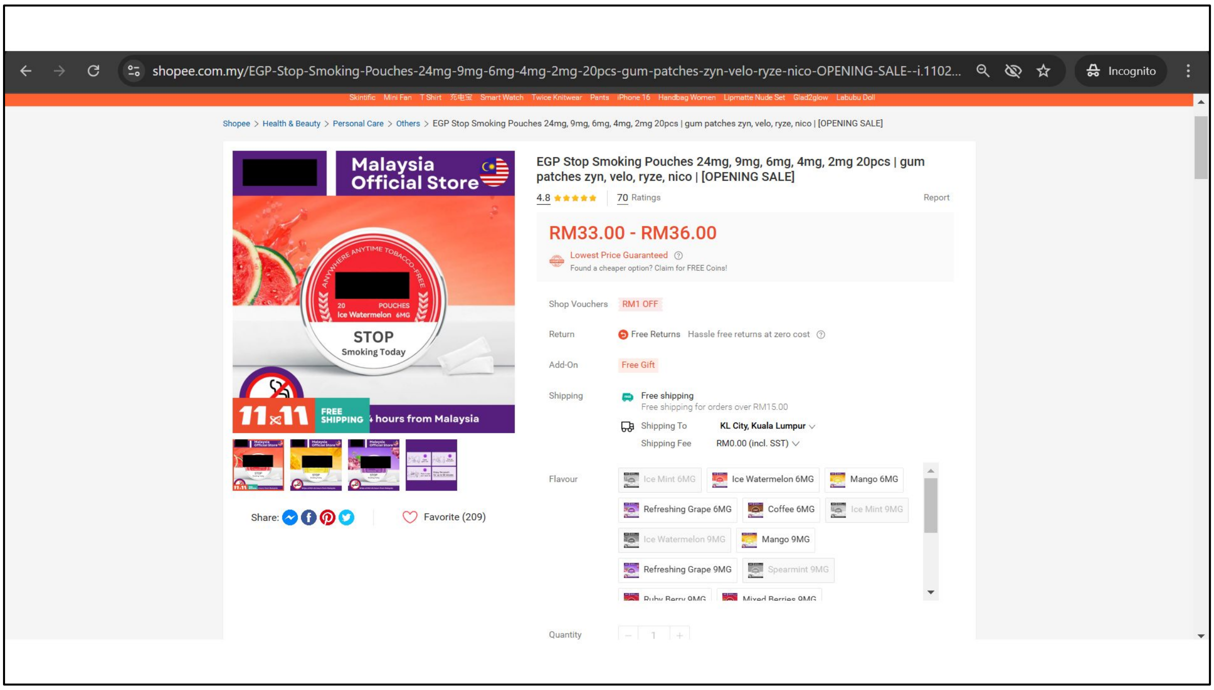

PRODUCT 1 - EGP Stop Smoking Pouches 24mg, 9mg, 6mg, 4mg, 2mg 20pcs | gum patches zyn, velo, ryze, nico | [OPENING SALE]

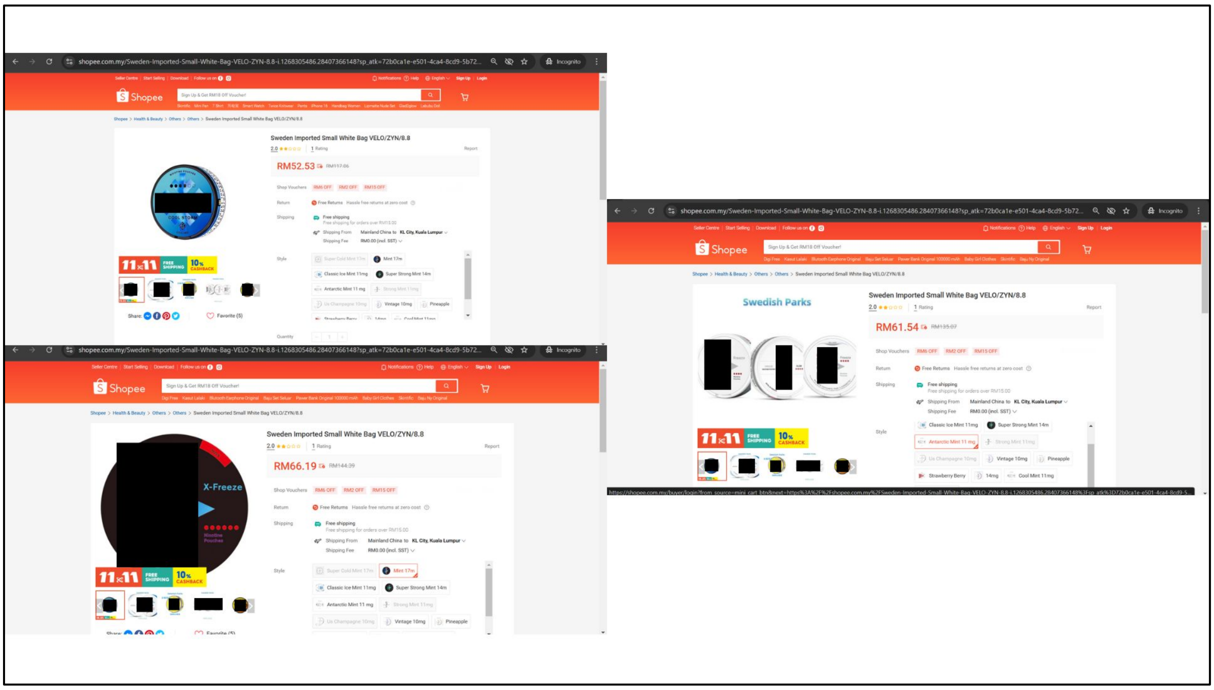

PRODUCT 2 - Sweden Imported Small White Bag VELO/ZYN/8.8

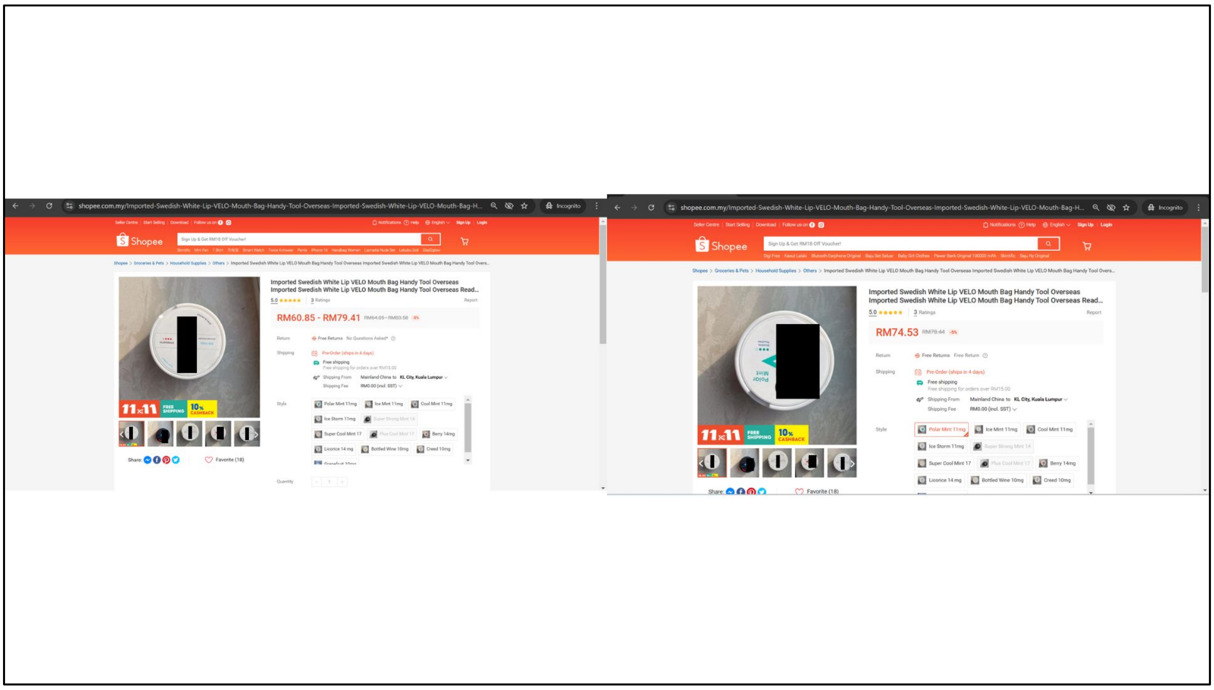

PRODUCT 3 - Imported Swedish White Lip VELO Mouth Bag Handy Tool Overseas

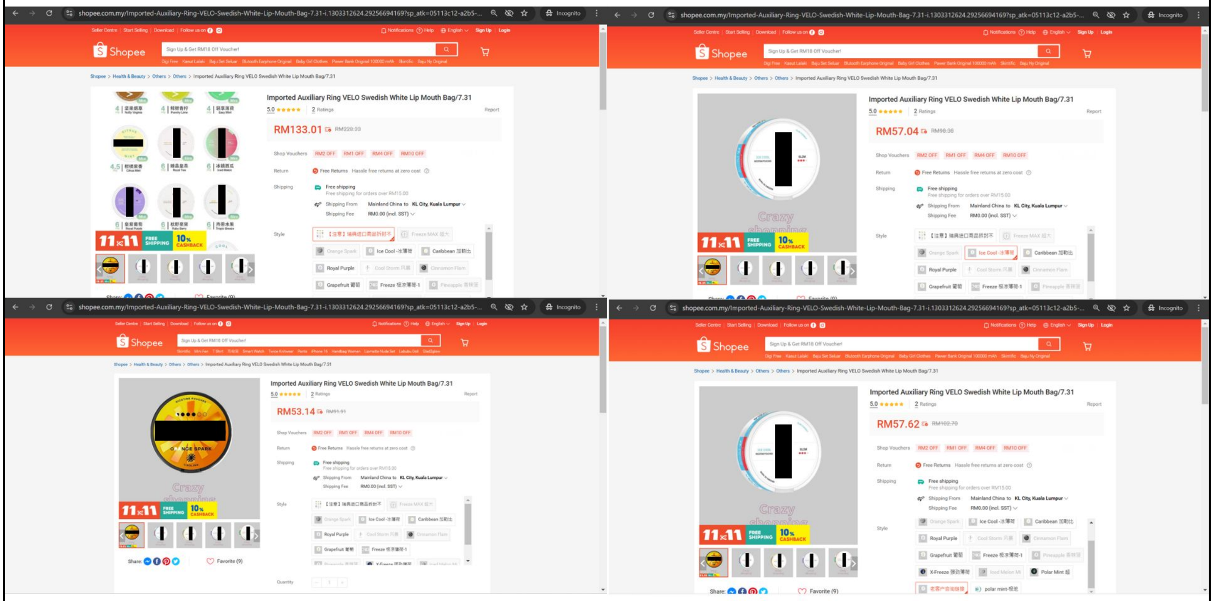

PRODUCT 4 - Imported Auxiliary Ring VELO Swedish White Lip Mouth Bag/7.31

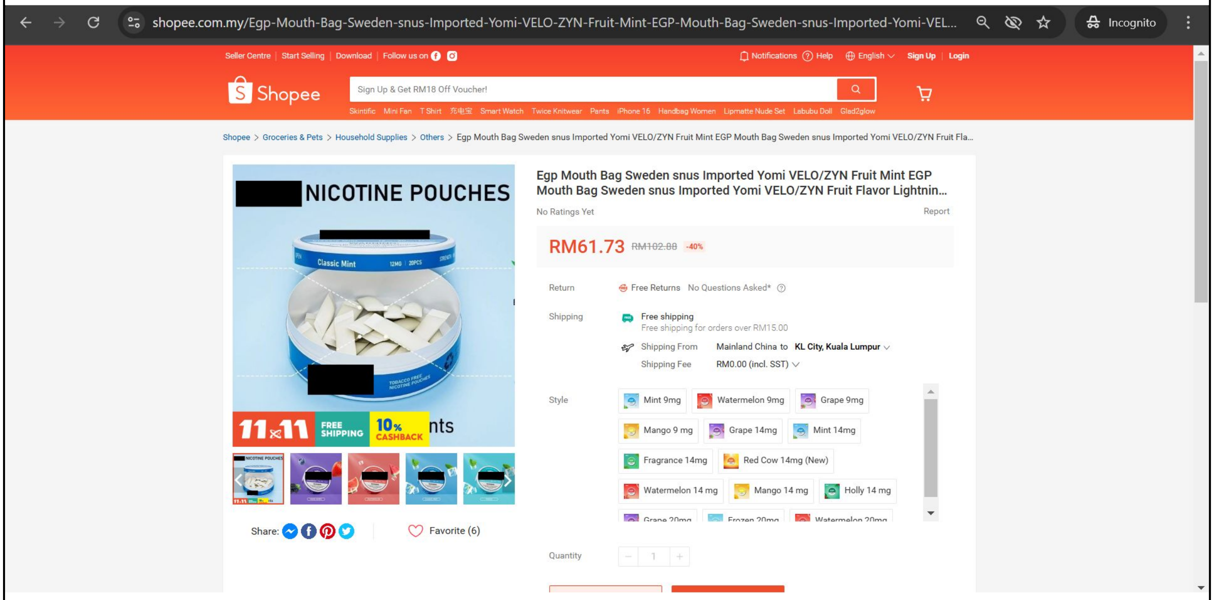

PRODUCT 5 - Egp Mouth Bag Sweden snus Imported Yomi VELO/ZYN Fruit Mint EGP Mouth Bag Sweden snus Imported Yomi VELO/ZYN Fruit Flavor Lightning

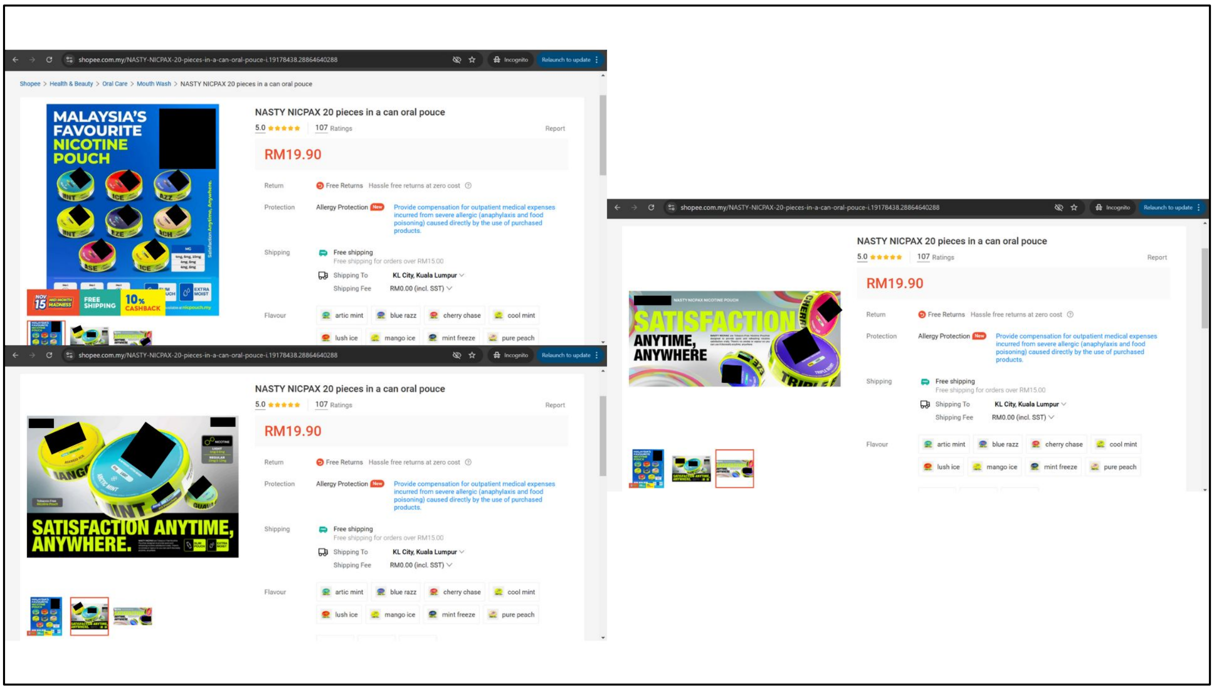

PRODUCT 7 - NASTY NICPAX 20 pieces in a can oral pouce

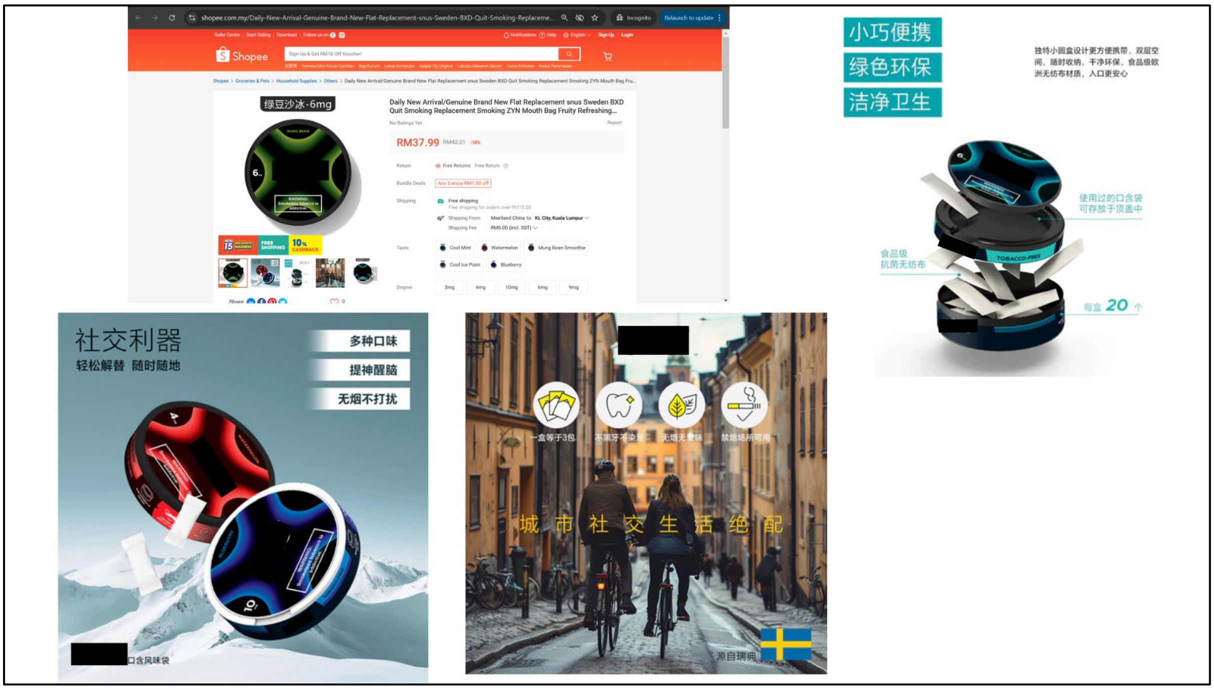

PRODUCT 8 - Daily New Arrival/Genuine Brand New Flat Replacement snus Sweden BXD Quit Smoking Replacement Smoking ZYN Mouth Bag Fruity Refreshing

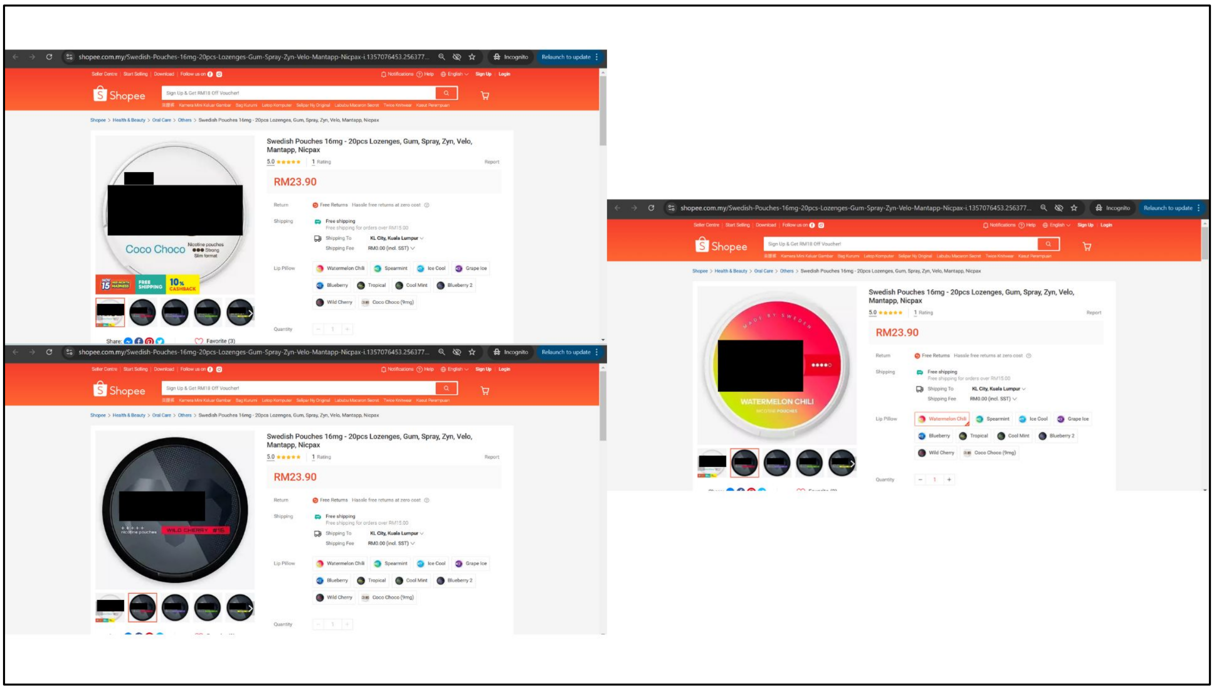

PRODUCT 9 - Swedish Pouches 16mg - 20pcs Lozenges, Gum, Spray, Zyn, Velo, Mantapp, Nicpax

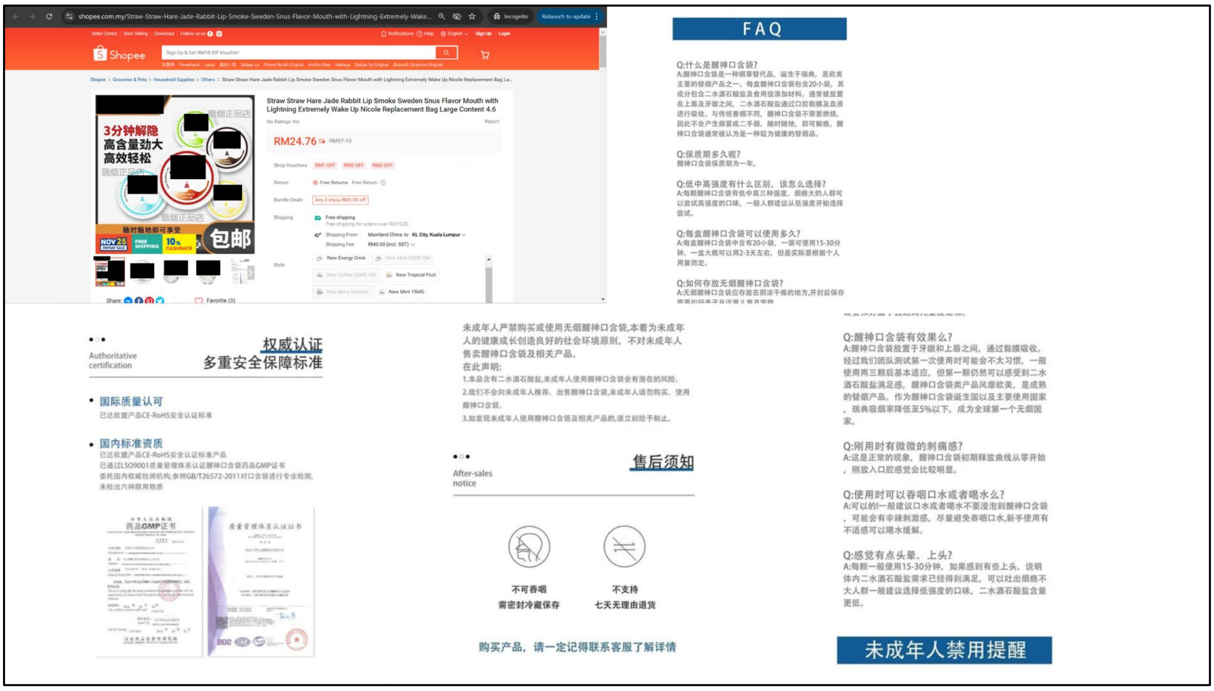

PRODUCT 10 - Straw Straw Hare Jade Rabbit Lip Smoke Sweden Snus Flavor Mouth with Lightning Extremely Wake Up Nicole Replacement Bag Large Content 4.6

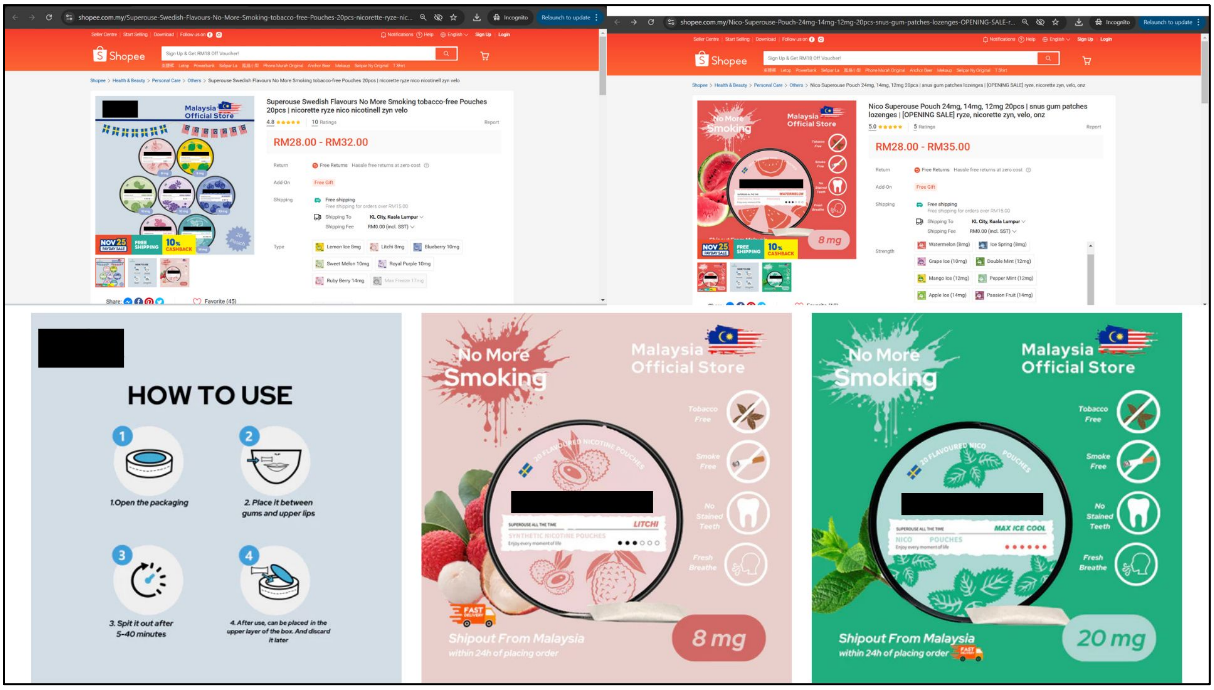

PRODUCT 11 - Superouse Swedish Flavours No More Smoking tobacco-free Pouches 20pcs | nicorette ryze nico nicotinell zyn velo

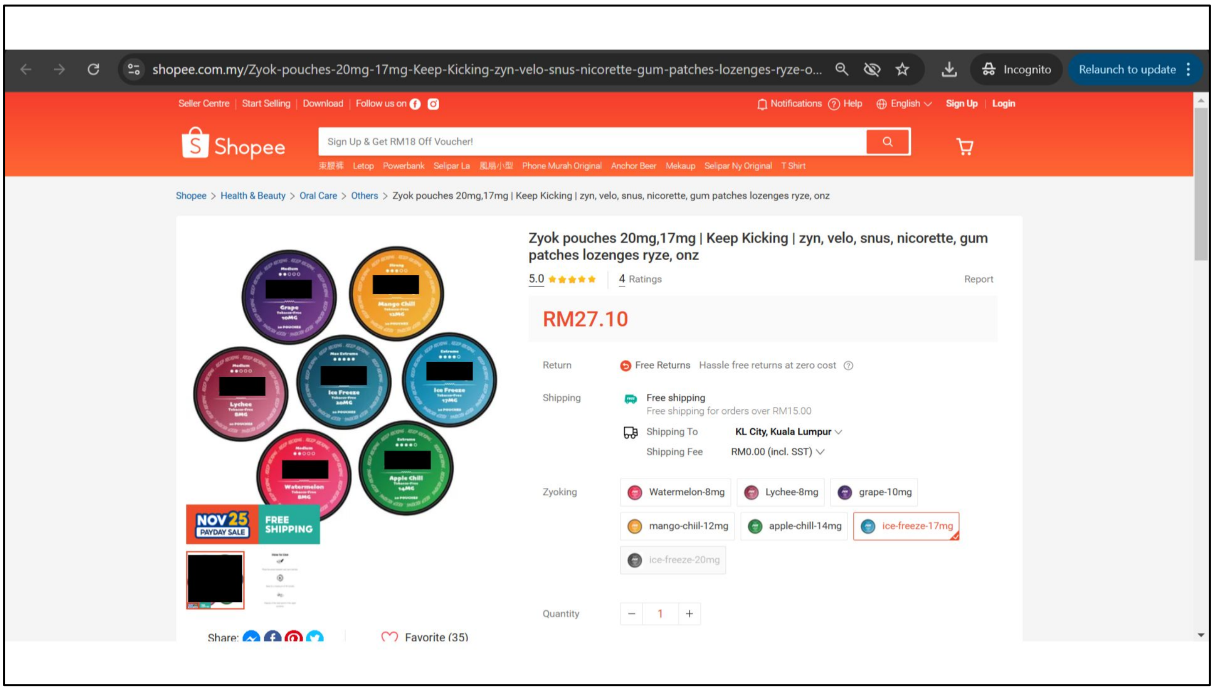

PRODUCT 12 - Zyok pouches 20mg,17mg | Keep Kicking | zyn, velo, snus, nicorette, gum patches lozenges ryze, onz

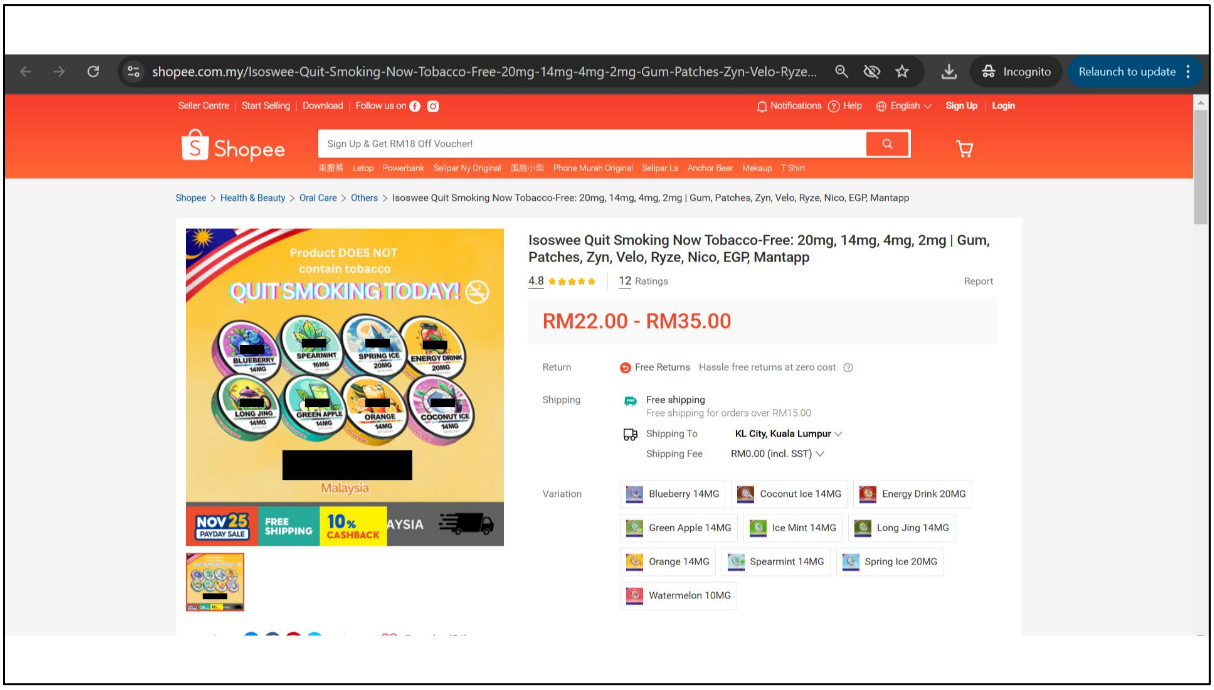

PRODUCT 13 - Isoswee Quit Smoking Now Tobacco-Free: 20mg, 14mg, 4mg, 2mg | Gum, Patches, Zyn, Velo, Ryze, Nico, EGP, Mantapp

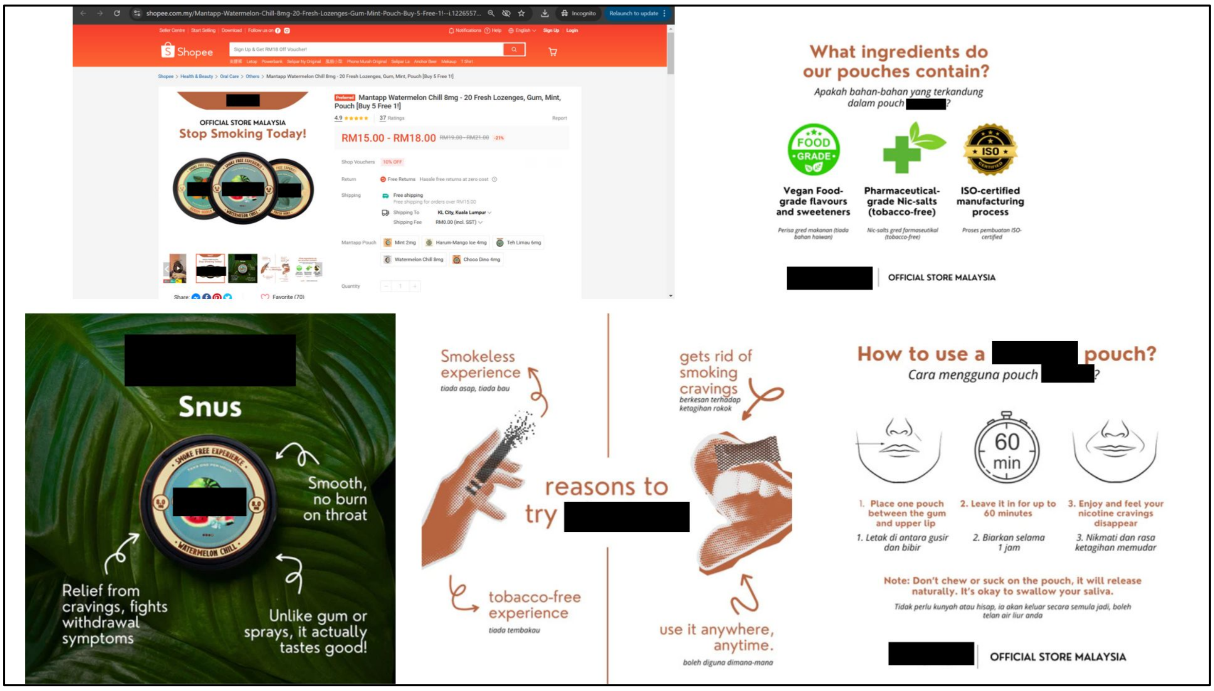

PRODUCT 14 - Mantapp Watermelon Chill 8mg - 20 Fresh Lozenges, Gum, Mint, Pouch [Buy 5 Free 1!]

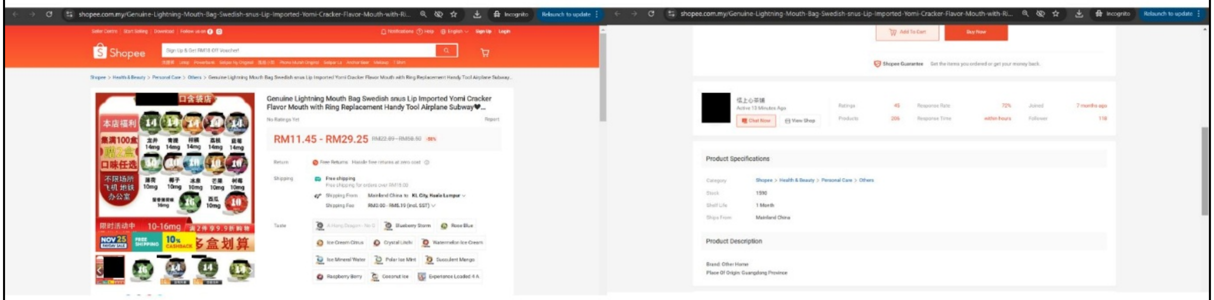

PRODUCT 15 - Genuine Lightning Mouth Bag Swedish snus Lip Imported Yomi Cracker Flavor Mouth with Ring Replacement Handy Tool Airplane Subway❤

ADDITIONAL PRODUCTS GLOBAL STORES

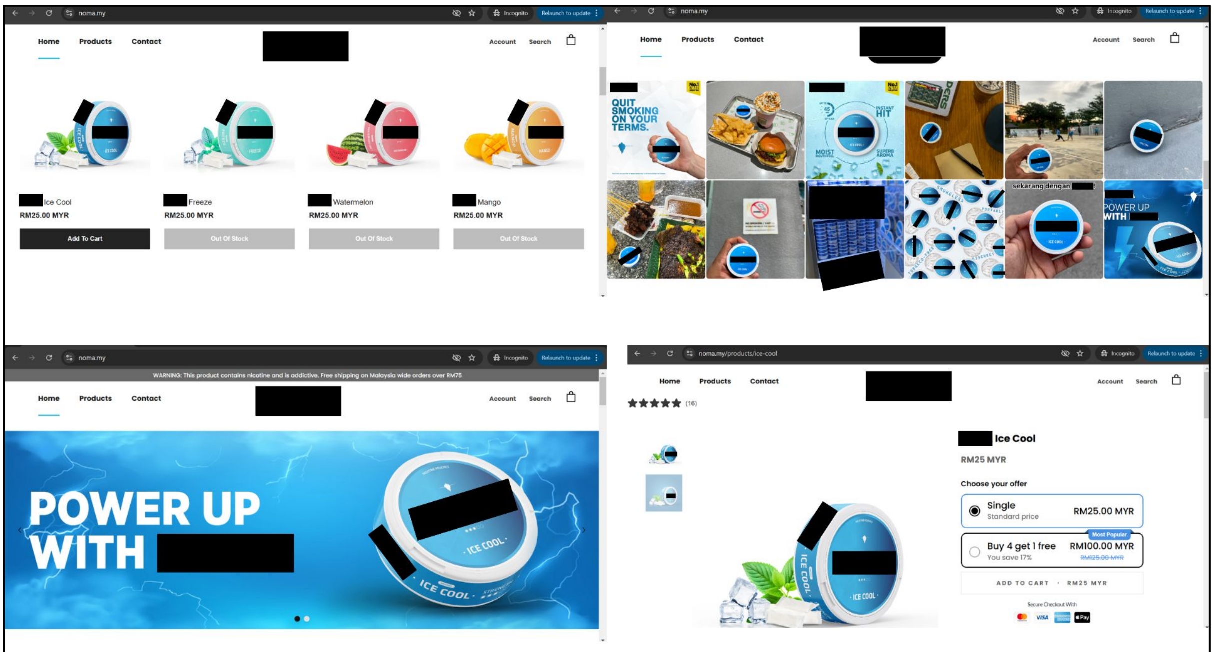

PRODUCT 1 - NOMA

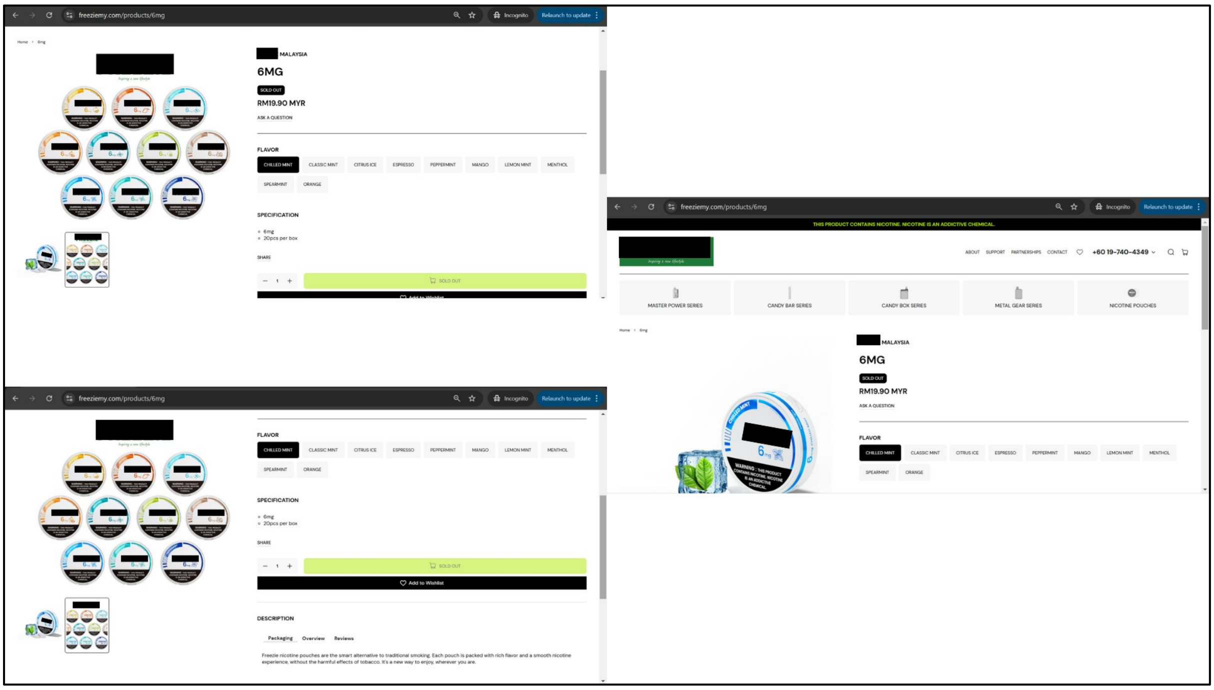

PRODUCT 2 - FREEZIE

© 2026 Sreeramareddy C.T. et al.
